# Analysis of herpes simplex type 1 gB, gD, and gH/gL on production of infectious HIV-1: HSV-1 gD restricts HIV-1 by exclusion of HIV-1 Env from maturing viral particles

**DOI:** 10.1186/s12977-019-0470-5

**Published:** 2019-04-02

**Authors:** Sachith Polpitiya Arachchige, Wyatt Henke, Maria Kalamvoki, Edward B. Stephens

**Affiliations:** Department of Microbiology, Molecular Genetics and Immunology, University of Kansas Medical Center, 2000 Hixon Hall, 3901 Rainbow Blvd., Kansas City, KS 66160 USA

**Keywords:** HSV-1, gD, gB, gH/gL, HIV-1, gp120/gp41, Env incorporation, Virus restriction

## Abstract

**Background:**

We previously showed that the gM of HSV-1 could restrict the release of infectious HIV-1 from cells. In this study, we analyzed if the four HSV-1 glycoproteins (gD, gB, and gH/gL), which are the minimum glycoproteins required for HSV-1 entry, restricted the release of infectious HIV-1.

**Results:**

Of these four glycoproteins, gD and gH/gL restricted the production of infectious HIV-1 from cells transfected with an infectious molecular clone of HIV-1 (strain NL4-3) while gB had no significant effect. Pulse-chase analyses indicated that gD did not affect the biosynthesis and processing of gp160 into gp120/gp41, the transport of the gp120/gp41 to the cell surface, or the release of HIV-1 particles from the cell surface. Our analyses revealed that gD was incorporated into HIV-1 virus particles while gp120/gp41 was excluded from released virus particles. Truncated mutants of gD revealed that the cytoplasmic domain was dispensable but that a membrane bound gD was required for the restriction of release of infectious HIV-1. Finally, cell lines expressing gD also potently restricted the release of infectious virus.

**Conclusions:**

Due to its ability to exclude HIV-1 gp120/gp41 from maturing virus, gD may provide a useful tool in deciphering mechanisms of Env incorporation into maturing virus particles.

**Electronic supplementary material:**

The online version of this article (10.1186/s12977-019-0470-5) contains supplementary material, which is available to authorized users.

## Background

Human herpes virus 1 (HHV-1 or HSV-1) encodes for at least 13 envelope glycoproteins, several of which are incorporated into mature virions. Of these viral glycoproteins, four glycoproteins (gB, gD and the heterodimer gH/gL) have been implicated in HSV fusion and entry [1–3]. The virus initially attaches to cells through the interaction of gB with heparan sulfate moieties of surface proteoglycans (HSPGs) [4]. However, gB binding to HSPG is insufficient for entry and requires the interaction between the receptor binding protein gD and either one of three specific cell surface receptors: (a) herpesvirus entry mediator (HVEM); (b) nectin-1; or (c) heparan sulfate specifically modified by 3-O-sulfotransferases [5–14]. The latter event triggers the membrane fusion process that is mediated by gB and gH/gL [2, 15, 16]. Importantly, the gB trimer and gH/gL heterodimer are conserved across the Family Herpesviridae while the receptor-binding gD varies among the family members.

Previously, we screened nine HSV-1 proteins for the ability to restrict the production of infectious HIV-1 [17]. We found that the HSV-1 gM protein restricted infectious virus production through a mechanism that prevented efficient processing of gp160 into gp120/gp41 and cell surface transport [17]. We further showed that deletion of the C-terminal region of gM (gDΔ345–473) abrogated the ability to restrict HIV-1 [17]. Here, we have assessed whether the four HSV-1 glycoproteins required for HSV-1 entry (gB, gD, gH/gL) would restrict the production of infectious HIV-1. We report that HSV-1 gD and gH/gL but not gB restricted the release of infectious virus. HSV-1 gD and HSV-2 gD (gD2) did not significantly alter the processing of gp160 and transport of gp120/gp41 to the cell plasma membrane. But rather, we observed that both gD molecules were incorporated into budding HIV-1 particles while excluding gp120/gp41. These results show that different HSV-1 membrane proteins can prevent the release of infectious HIV-1 through different mechanisms. Finally, we show that cell lines stably expressing gD potently restricted the release of HIV-1 verifying the results of our transfection studies.

## Results

### The HSV-1 gD and gH/gL but not gB restricts the release of infectious HIV-1 from cells

**Fig. 1:**
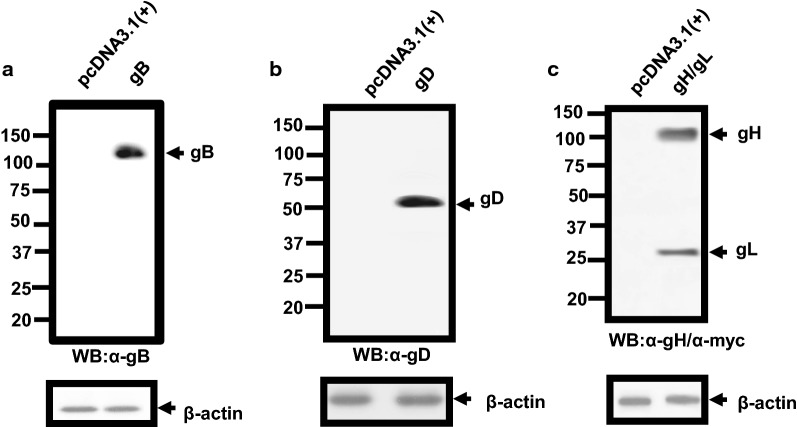
Expression of HSV-1 glycoproteins. 293 cells were transfected with plasmids expressing HSV-1 gB, gD, and gH/gL. At 24 h, cells were lysed in RIPA buffer and analyzed for protein expression by immunoblot analysis using antibodies against gB (**a**), gD (**b**) and gH and anti-myc for gL (**c**)

**Fig. 2:**
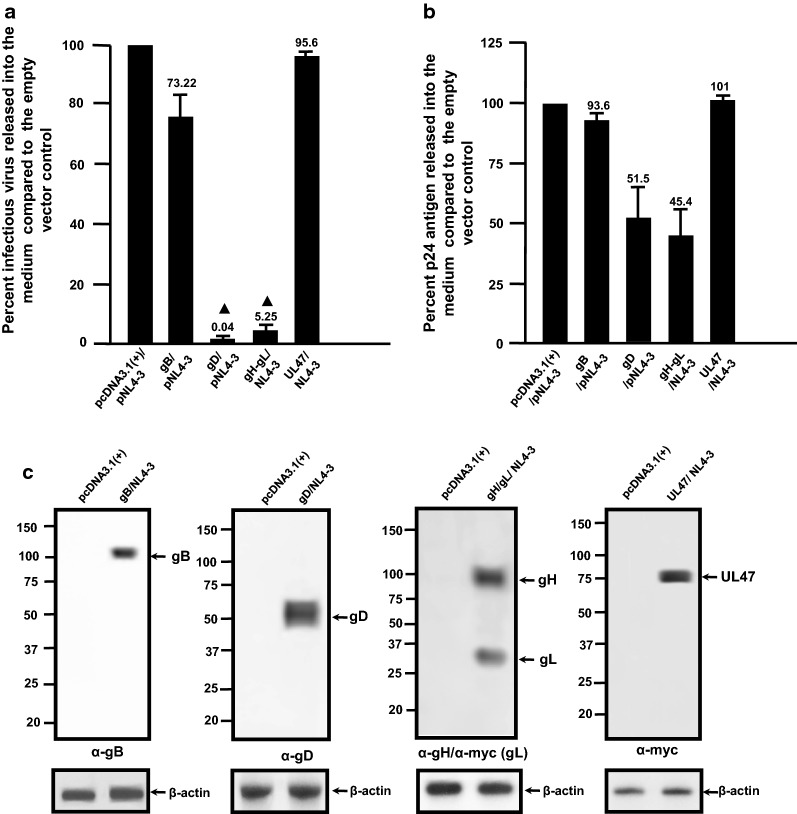
HSV-1 gD and gH/gL but not gB restricts the production of infectious HIV-1. 293 cells were co-transfected with either the empty pcDNA3.1(+) plasmid or plasmids expressing HSV-1 gD, gB, gH/gL, or  UL47 proteins and pNL4-3. At 48 h, the culture supernatants were collected. The levels of infectious virus released into the culture supernatants was determined using TZM-bl cell assays and p24 in the culture supernatants determined using p24 antigen capture assays. **a** The level of infectious virus released into the culture medium from cells co-transfected with pcDNA3.1(+) or a plasmid expressing gD, gB, gH/gL, or UL47 and pNL4-3. **b** The levels of p24 protein produced from cells co-transfected with either the empty pcDNA3.1(+) plasmid or expressing gD, gB, gH/gL, or UL47 proteins and pNL4-3. **c** The cell lysates from the above restriction assays were analyzed for the presence of gD, gB, gH/gL, or UL47 using immunoblots and appropriate antibodies. The experiments were performed at least four times and statistical differences with the pcDNA3.1(+)/HIV-1 control evaluated using a two-tailed Student’s *t*-test, with *p *< 0.01 (filled triangle) considered significant

We first analyzed the expression of gB, gD, and gH/gL in the absence of the HIV-1 genome. For these experiments, we chose to express gH and gL together as previous studies have shown expression of both proteins are necessary for optimal folding and transport to the cell surface, otherwise the proteins are unstable [18, 19]. 293 cells were transfected with the empty pcDNA3.1(+) vector or one expressing HSV-1 gB, gD, or gH/gL. At 24 h post-transfection, cells were lysed and HSV-1 proteins examined by immunoblot analysis. The results show that HSV-1 gB, gD and gH/gL were detected in cell lysates (Fig. 1). We next performed restriction assays in which 293 cells were co-transfected with empty pcDNA3.1(+) vector or one expressing either HSV-1 gB, gD, or gH/gL glycoproteins and pNL4-3. At 48 h, the culture medium was harvested and assayed for the presence of infectious virus using TZM-bl assays and p24 as described in the Methods section [20–23]. Cell lysates were prepared and used in Western blots to detect the HSV-1 proteins. Our results indicate that in the presence of gB, gD, or gH/gL, the level of infectious virus released was 73.2%, 0.04%, and 5.25% of the pcDNA3.1(+) control, respectively (Fig. 2a). As a control, we also transfected cells with HSV-1 UL47, which we previously showed did not restrict HIV-1 [17]. Expression of this protein did not restrict the production of infectious HIV-1 (Fig. 2a). Examination of the p24 levels released from transfected cells indicate that p24 was released at 93.6%, 51.5%, 45.4% and 101% for gB, gD, gH/gL, and UL47, respectively, when compared to the pcDNA3.1(+) control (Fig. 2b). Finally, all HSV-1 proteins were expressed well in cells from the co-transfections with pNL4-3 (Fig. 2c). Taken together these results indicate that there was a profound restriction of infectious HIV-1 production in the presence of HSV-1 gD and gH/gL but not the gB glycoprotein. The finding that gD potently restricted the release of infectious HIV-1 while gB had no effect suggests that the results were not an overexpression artifact. We also performed an immunoprecipitation experiment comparing the level of gD expression in 293 cells transfected with same amount of plasmid used in the above experiments to  293 cells infected with HSV-1 with an M.O.I. of 0.1. The results of this experiment showed that the levels of gD in 293 cells infected with HSV-1 were less than with the transfected cells (Additional file 1: Fig. S1).

### HSV-1 gB, gD and gH/gL do not significantly affect HIV-1 viral proteins synthesis

**Fig. 3:**
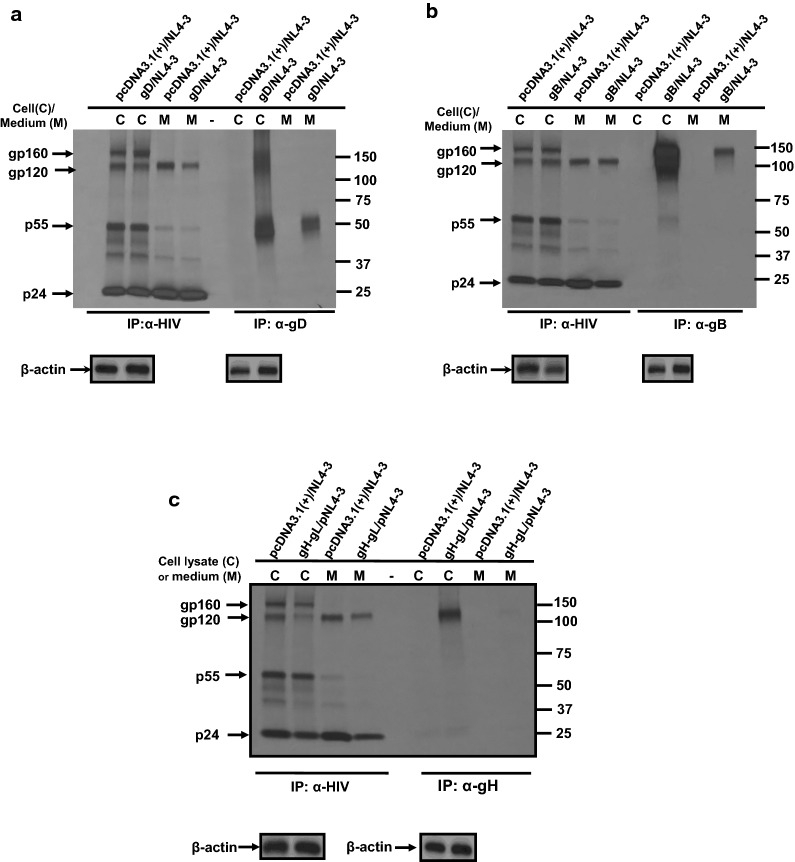
HSV-1 gB, gD, and gH/gL do not significantly affect the synthesis and processing of HIV-1 viral proteins. 293 cells were transfected with either the empty pcDNA3.1(+) vector or one expressing HSV-1 gB, gD, or gH/gL and pNL4-3. At 30 h post-transfection, cells were starved for methionine/cysteine and radiolabeled with ^35^S-methionine/cysteine for 16 h. The culture medium was collected and cell lysates prepared as described in the Materials and Methods section. HIV-1 proteins were immunoprecipitated using anti-HIV-1 antibodies while HSV-1 proteins were immunoprecipitated with gB, gD, gH, or myc (gL) antibodies. The immunoprecipitates were collected on protein-A-Sepharose, washed, and boiled in sample reducing buffer. The proteins were separated on 7.5% SDS-PAGE and visualized using standard radiographic techniques. **a** Immunoprecipitation of HIV-1 and gD proteins from cell lysates or culture medium from 293 cells co-transfected with either pcDNA3.1(+) and pNL4-3 or a vector expressing gD and pNL4-3. **b** Immunoprecipitation of HIV-1 and gB proteins from cell lysates or culture medium from 293 cells co-transfected with either pcDNA3.1(+) and pNL4-3 or a vector expressing gB and pNL4-3. **c** Immunoprecipitation of HIV-1 and gH/gL proteins from cell lysates or culture medium from 293 cells co-transfected with vectors pcDNA3.1(+) and pNL4-3 or vectors expressing gH/gL and pNL4-3. Shown below each cell lysate panel is the expression of β-actin as a loading control

We analyzed the biosynthesis by metabolic labeling of HIV-1 proteins in the presence of gB, gD, and gH/gL. 293 cells were transfected with either pcDNA3.1(+) or one expressing gD and pNL4-3. At 30 h post-transfection, cells were starved for methionine/cysteine and radiolabeled with ^35^S-methionine/cysteine for 16 h. The culture medium was harvested and cell lysates prepared as described in the Methods section. HIV-1 proteins were immunoprecipitated using anti-HIV-1 antibodies. HSV-1 gD proteins immunoprecipitated using an anti-gD monoclonal antibody. In the presence of the empty vector, HIV-1 gp160, gp120, gp41, p55 Gag and p24 proteins were detected in the cell lysates and gp120 and p24 were predominantly detected in the culture medium with p55 Gag and gp41 also detected at lower levels (Fig. 3a). In the presence of gD, the same HIV-1 proteins were detected in both the cell lysates and culture medium at approximately the same levels, although we consistently observed that the level of gp120 released from cells at slightly reduced levels. The gD was easily detectable in cell lysates and the culture medium from gD/pNL4-3 co-transfected cells (Fig. 3a). Co-transfection of cells with gB/pNL4-3 resulted in similar levels of HIV-1 proteins expressed, processed and released into the culture medium and with gB detected in both the cell lysates and culture medium (Fig. 3b). Finally, co-transfection of cells with gH/gL and pNL4-3 resulted in essentially the same profile as co-transfection with gD/pNL4-3 except that gH was not detected in the culture medium (Fig. 3c). Taken together, these results indicate that in the presence of gD, gB, or gH/gL, there were no significant differences in the biosynthesis and processing of the HIV-1 Gag and Env proteins.

### The presence of HSV-1 gD and gB does not alter the kinetics of gp160 processing and transport to the cell plasma membrane

**Fig. 4:**
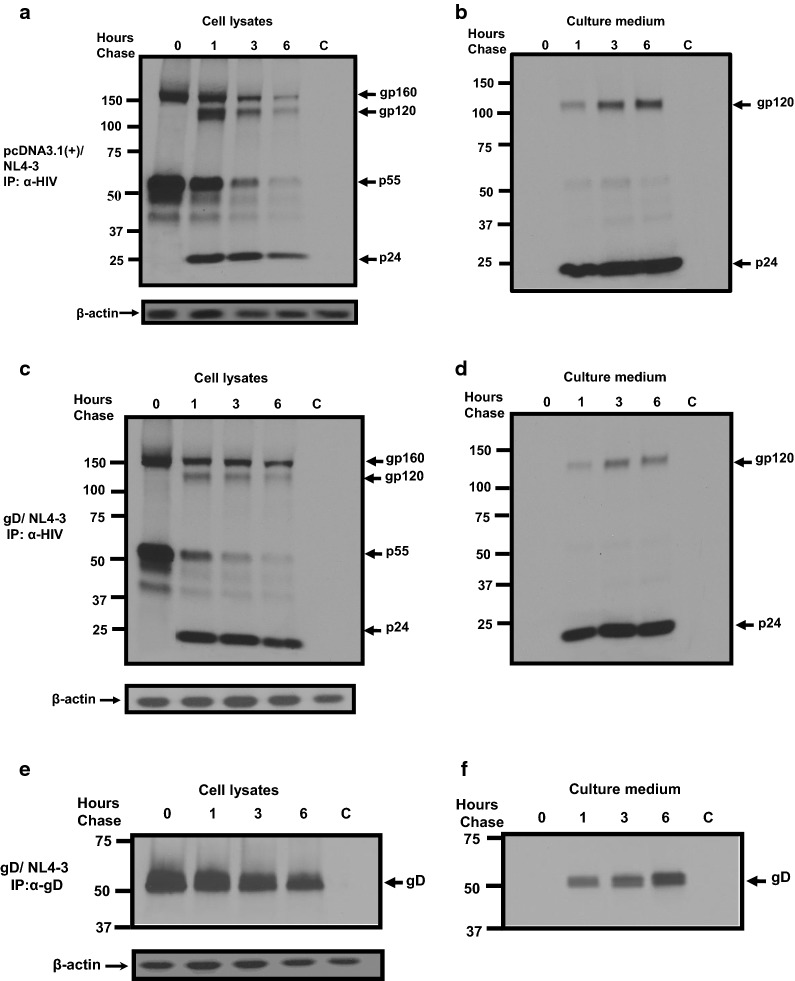
HSV-1 gD and gB do not significantly affect the processing of HIV-1 Gag and gp160. 293 cells were co-transfected with empty pcDNA3.1(+) vector or one expressing gD and pNL4-3. At 24 h, cells were starved in medium lacking methionine/cysteine for 2 h followed by radiolabeling cultures with ^35^S-methionine/cysteine. The radiolabel was removed and washed three times in medium containing 100X methionine/cysteine and chased in the same medium for 0, 1, 3, and 6 h. The culture medium was harvested and cell lysates prepared as described in the Materials and Methods. HIV-1 Env and Gag proteins were immunoprecipitated using the HIV-1 antibodies and HSV-1 gD was immunoprecipitated with an appropriate monoclonal antibody. The immunoprecipitates were collected on protein-A-Sepharose beads, washed, and boiled in sample reducing buffer. The proteins were separated on 7.5% SDS gels and visualized using standard radiographic techniques. **a**, **b** HIV-1 proteins immunoprecipitated from the cell lysates (**a**) and culture medium (**b**) of cells co-transfected with pcDNA3.1(+) and pNL4-3. **c**, **d** HIV-1 proteins immunoprecipitated from the cell lysates (**c**) and culture medium (**d**) of cells co-transfected with a vector expressing gD and pNL4-3. **e**, **f** HSV-1 gD protein immunoprecipitated from cell lysates (**e**) and culture medium (**f**) of cells co-transfected with a vector expressing gD and pNL4-3

As gD showed the highest level of restriction of infectious HIV-1 release, we further characterized the mechanism of gD restriction. We performed pulse-chase analyses to examine the synthesis and processing of HIV-1 proteins in the presence of HSV-1 gD or gB (as a negative control). 293 cells were transfected with either the empty pcDNA3.1(+) vector or one expressing HSV-1 gD or gB and the pNL4-3 vector. At 24 h post-transfection, cells were starved for methionine/cysteine and radiolabeled with ^35^S-methionine/cysteine, and chased for 0, 1, 3, and 6 h in medium with cold excess methionine and cysteine. Co-transfection of 293 cells with pcDNA3.1(+) and pNL4-3 resulted in the HIV-1 gp160 and Gag precursors synthesized and processed into gp120 and p24 by the 1 h chase period (Fig. 4a). Immunoprecipitation of HIV-1 proteins from the culture medium indicate that gp120 and p24 were detected in the culture medium beginning at the 1 h chase period, which increased in levels during the 3 and 6 h chase periods (Fig. 4b). The kinetics of biosynthesis and processing of Gag and Env proteins were similar in pulse-chase analysis of cells co-transfected with gD and pNL4-3 (Fig. 4c, d). However, we consistently found that the levels of gp160 precursor and cleavage product gp120 were slightly reduced in cell lysates in gD/pNL4-3 cultures when compared to pcDNA3.1(+)/pNL4-3. Analysis of gD expression from the same lysates and medium indicate that gD was expressed and released into the culture medium (Fig. 4e, f). Analysis of cells co-transfected with gB and pNL4-3 also revealed similar processing and release of HIV-1 proteins and that gB was also released into the culture medium (Additional file 2: Fig. S2). Taken together, these results indicate that the kinetics of biosynthesis and processing of HIV-1 Env and Gag were not significantly affected by the presence of HSV-1 gD or gB and suggests that both proteins were released from cells.

### HSV-1 gD and gB are released from HIV-1 transfected cells and incorporated into HIV-1 particles but only gD prevents the incorporation of gp120/gp41 into particles

**Fig. 5:**
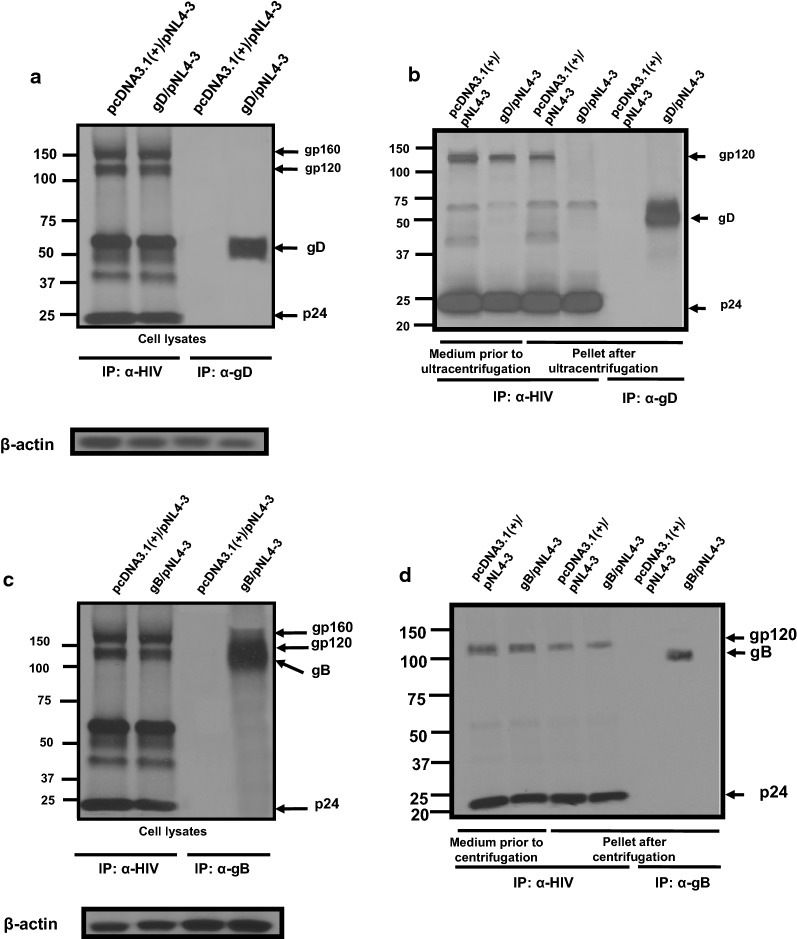
HSV-1 gD and gB are efficiently incorporated into HIV-1 particles. 293 cells were co-transfected with either empty pcDNA3.1(+) vector, one expressing the HSV-1 gD or HSV-1 gB and pNL4-3. At 30 h, the cells were starved for methionine/cysteine for 2 h and then radiolabeled for with ^35^S-methionine/cysteine for 16 h. At 48 h post-transfection, the cell culture medium was harvested and subjected to low speed centrifugation to remove cellular debris and virus pelleted through a 20% sucrose cushion using ultracentrifugation. The cells were lysed in RIPA buffer on ice, centrifuged to remove the nuclei. The resulting supernatant was transferred to a new microfuge tube. The medium, cell lysates, and virus pellet  were used in immunoprecipitation analysis using anti-HIV-1 antibodies (to immunoprecipitate Env and Gag proteins) and appropriate monoclonal antibodies to immunoprecipitate HSV-1 gD and gB. The immunoprecipitates were collected on protein-A-Sepharose beads, washed, and boiled in sample reducing buffer. The proteins were separated on 7.5% SDS-PAGE and visualized using standard radiographic techniques. **a** HIV-1 and gD proteins immunoprecipitated from cell lysates prepared from cells co-transfected with either empty vector and pNL4-3 or a vector expressing gD and pNL4-3. **b** Cells were co-transfected with either empty vector and pNL4-3 or a vector expressing gD and pNL4-3 followed by immunoprecipitation of HIV-1 and gD proteins from the culture medium and pellet after ultracentrifugation. **c** HIV-1 and gB proteins immunoprecipitated from cell lysates prepared from cells co-transfected with either empty vector and pNL4-3 or a vector expressing gB and pNL4-3. **d** Cells were co-transfected with either empty vector and pNL4-3 or a vector expressing gB and pNL4-3 followed by immunoprecipitation of HIV-1 and gB proteins from the culture medium and pellet after ultracentrifugation

**Fig. 6:**
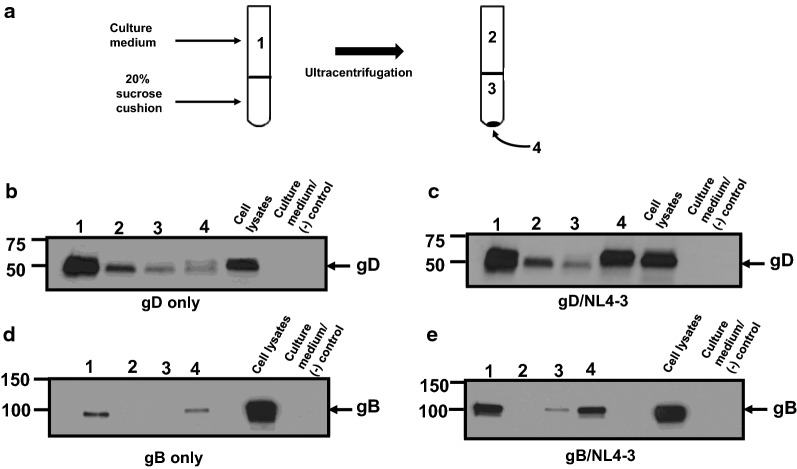
Both gD and gB are more efficiently released in presence of virus. In companion cultures from the experiment in Fig. 5, 293 were co-transfected with either 1) the empty pcDNA3.1(+) vector; 2) the vector expressing gD; 3) the vector expressing gD and pNL4-3; 4) the vector expressing gB; or 5) the vector expressing gB and pNL4-3. The cells were radiolabeled and harvested exactly as in Fig. 5. Following low speed centrifugation, the culture supernatants were layered onto a 20% sucrose cushion and virus pelleted by ultracentrifugation and different fractions were analyzed for the presence of gD or gB using immunoprecipitation. **a** Schematic showing the fractions analyzed: Fraction 1: medium prior to ultracentrifugation; Fraction 2: medium following ultracentrifugation; Fraction 3: the sucrose cushion following ultracentrifugation; and Fraction 4: virus pellet. **b** Immunoprecipitation of gD proteins from the different fractions of cells transfected with vector expressing gD. **c** Immunoprecipitation of gD proteins from the different fractions of cells co-transfected with the vector expressing gD and pNL4-3. **d** Immunoprecipitation of gB proteins from the different fractions of cells transfected with gB. **e** Immunoprecipitation of gB proteins from the different fractions of cells co-transfected with the vector expressing gB and pNL4-3. The amount of immunoprecipitated proteins run on gels represent 25% (Fraction 1), 25% (Fraction 2), 100% (Fraction 3) and 50% (Fraction 4) of the total immunoprecipitated product

As we observed that HSV-1 gD was released into the culture medium of cells co-transfected with HIV-1, we next determined if gD was incorporated into virus particles. 293 cells were transfected with either the pcDNA3.1(+) vector, one expressing HSV-1 gD, or gB, and pNL4-3 plasmid. At 30 h post-transfection, the medium removed, cells starved for methionine/cysteine and radiolabeled with ^35^S-methionine/cysteine for 16 h. The culture medium was harvested and cell lysates prepared. The culture medium was clarified by low speed centrifugation, the supernatant collected and overlaid onto a 20% sucrose cushion and virus pelleted. The supernatant and pellet fractions were collected and analyzed for the presence of gp120, Gag p24, gD and gB by immunoprecipitation analysis. Co-transfection of cells with pcDNA3.1(+) or gD and pNL4-3 revealed that the HIV-1 proteins and gD were present in the cell lysates (Fig. 5a). Immunoprecipitation of proteins from the culture medium and viral pellet of cells co-transfected with empty vector and pNL4-3 revealed the presence of gp120 and p24 in the culture medium (Fig. 5b). Transfection of cells with the vectors expressing gD and pNL4-3 also resulted in the release of gp120, p24 and gD in the culture medium but gp120 was significantly reduced in the viral pellet (Fig. 5b). The gD protein was also detected in the virus pellet (Fig. 5b). Co-transfection of cells with the vectors expressing gB and pNL4-3 also resulted in the release of gp120, p24 and gB into the culture medium and viral pellet (Fig. 5c, d). Similarly, HSV-1 gB was also detected in the culture medium and the virus pellet (Fig. 5c, d). Taken together, these results suggest that gD and gB were likely incorporated into virus. We next layered the culture medium over a 20% sucrose cushion and pelleted the virus by ultracentrifugation and analyzed the upper medium fraction, the sucrose cushion, and the pellet fractions for the presence of gD or gB by immunoprecipitation. A schematic describing the fractions analyzed is shown in Fig. 6a. As a control we also analyzed the medium from cells transfected with vectors expressing gD or gB. The results indicate that both gD and gB were present in the culture medium prior to ultracentrifugation (Fig. 6b–e). For cells transfected with gD alone (Fig. 6b), the protein was still detectable in upper fraction (fraction 2), in the sucrose cushion (fraction 3) and small amounts were detected in the pellet (fraction 4). In cells co-transfected with gD and pNL4-3 (Fig. 6c), a similar pattern was observed except that higher levels of gD were immunoprecipitated from the pellet after ultracentrifugation (fraction 4). In cells transfected with the vector expressing gB alone (Fig. 6d), the protein was not detected in fractions 2 and 3 but was detectable in the pellet (fraction 4). Like gD/NL4-3, more gB was immunoprecipitated from the pellet from cells co-transfected with gB/pNL4-3 (Fig. 6e).

**Fig. 7:**
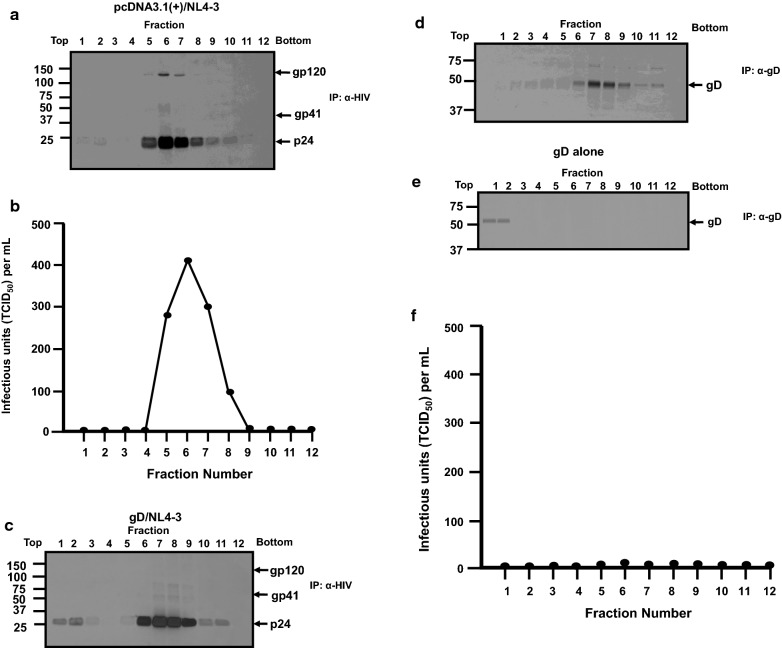
Sucrose density gradient centrifugation purification of virus reveals the gp120 is not incorporated in viral particles in the presence of HSV-1 gD. 293 cells were co-transfected with either empty pcDNA3.1(+) vector and pNL4-3, a vector expressing gD and pNL4-3, or a vector expressing gB and pNL4-3. At 30 h, the cells were starved for methionine/cysteine, radiolabeled and the culture medium harvested at 48 h post-transfection. Following low speed centrifugation, the culture supernatants were layered onto a 20% sucrose cushion and virus pelleted by ultracentrifugation. The pelleted virus resuspended in DMEM without serum and layered on a discontinuous 20–60% sucrose gradient. The virus was subjected to ultracentrifugation for 20 h (76,000 x g, SW55Ti rotor), 12 fractions were collected, and subjected to immunoprecipitation analysis using anti-HIV-1 antibodies to immunoprecipitated HIV-1 Gag and Env) and appropriate monoclonal antibodies to immunoprecipitate HSV-1 gD or gB. The immunoprecipitates were collected on protein-A-Sepharose, washed, and boiled in sample reducing buffer. The proteins were separated on 7.5% SDS-PAGE and visualized using standard radiographic techniques. **a** Immunoprecipitation of HIV-1 proteins from gradient fractions of cells co-transfected with empty pcDNA3.1(+) vector and pNL4-3. **b** Analysis of virus infectivity from various fractions in (**a**). **c** Immunoprecipitation of HIV-1 proteins from gradient fractions of cells co-transfected with a vector expressing gD and pNL4-3. **d** Immunoprecipitation of HSV-1 gD from gradient fractions of cells transfected with a vector expressing gD and pNL4-3. **e** Immunoprecipitation of gD from gradient fractions of cells transfected with a vector expressing gD. **f** Analysis of virus infectivity from various fractions in (**c**, **d**)

We expanded on the above observations by further purification of pelleted virus from above on 20–60% discontinuous sucrose gradients. Fractions were collected and analyzed for the presence of gD, and HIV-1 proteins. Purification of pelleted virus from cells transfected with pcDNA3.1(+) and pNL4-3 revealed that HIV-1 Gag p24 was detected in fractions 1–2 and 5–11 with the highest amounts detected in fractions 5–7 while gp120 was detected in fractions 5–7 (Fig. 7a). The detection of gp120 and p24 correlated nicely with analysis of infectivity of the different fractions (Fig. 7b). Purification of virus from cells transfected with the vector expressing gD and pNL4-3 revealed that p24 was detected in fractions 1–2 and 6–11 with highest levels detected in fractions 6–9. HIV-1 gp120 was not detected in gradient fractions (Fig. 7c). However, long exposures resulted in gp120 detected in fractions 1–2 (unpublished data). In the same gradients, gD was detected in fractions 4–11 with the highest amounts in fractions 6–9 (Fig. 7d). As a control for these studies, we transfected 293 cells with the vector expressing gD, harvested the culture medium and layered a 20% sucrose cushion and subjected it to ultracentrifugation. The resulting pellet was resuspended and layered onto a 20–60% sucrose gradient and subjected it to ultracentrifugation as described above. Analysis of the fractions by immunoprecipitation revealed that gD was detectable in fractions 1–2 (Fig. 7e) but was not detectable in fractions where virus was localized in cells transfected with pNL4-3 or pNL4-3/gD (Fig. 7a, b). These results indicate the gD that co-migrated with virus was likely due to its incorporation into virus. The lack of gp120 in fractions associated with virus (6–9) also correlated with a lack of virus infectivity (Fig. 7f). We also analyzed the virus pellet for the presence of the transmembrane protein gp41 using a monoclonal antibody directed against this protein. Our results indicate in the presence of gD, gp41 was in the cell lysates but not in the culture medium or pelleted virus, indicating that gD did not merely result in the shedding of gp120 from the virus particles (Additional file 3: Fig. S3). We also analyzed virus on gradients from cells co-transfected with a vector expressing gB and pNL4-3 (Additional file 4: Fig. S4). HIV-1 p24 and gp120 were detected in fractions 6–9 with the highest levels observed in Fraction 6–7 while the gB protein was detected in fractions 6–9 (Additional file 4: Fig. S4). These results indicate that both gD and gB co-sedimented with HIV-1 but gp120 was only observed in virus when cells were co-transfected with gB and pNL4-3. Finally, we isolated viral RNA isolated from sucrose gradient purified virus grown in the presence of gD. We amplified the *gag*, *pol*, *vpu, env*, and *nef* genes using RT-PCR. Our results indicated that these genes were intact (data not shown).

### Over-expression of HIV-1 Env and gD still results in gp120/gp41 exclusion from purified virus

**Fig. 8:**
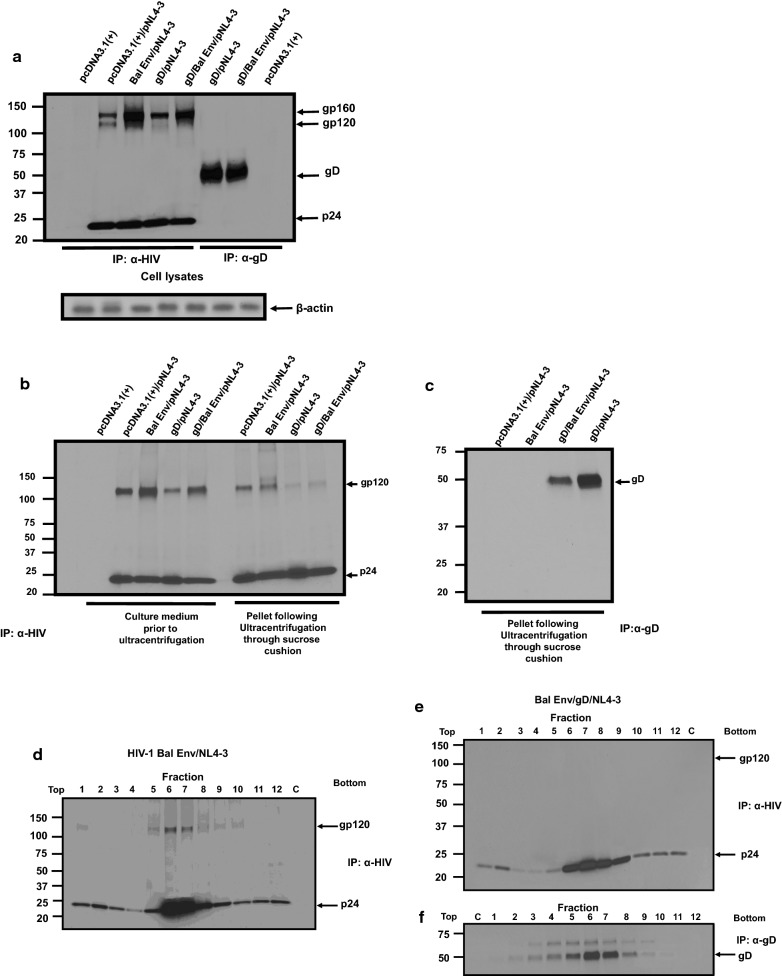
Over-expression of HSV-1 gD and HIV-1 Env still results in exclusion of HIV gp120/gp41 from particles. 293 cells were co-transfected with either the empty pcDNA3.1(+) vector and pNL4-3, a vector expressing Bal Env and pNL4-3, a vector expressing gD and pNL4-3, or vectors expressing gD, Bal Env and pNL4-3. At 30 h post-transfection, the cells were starved for 2 h with methionine/cysteine-free media and then radiolabeled for 16 h with ^35^S-methionine/cysteine. The culture media was harvested and subjected to low speed centrifugation to remove cellular debris. The resulting supernatant was layered on a 20% sucrose cushion and subjected to ultracentrifugation to pellet viral particle as described in the Materials and methods. The pelleted virus was harvested, resuspended in 200 μl of DMEM without serum and layered on a discontinuous 20-60% sucrose gradient. The virus was subjected to ultracentrifugation for 20 h at 76,000×*g* in a SW55Ti at which time the fractions were collected and proteins immunoprecipitated using anti-HIV-1 antibodies to immunoprecipitate HIV-1 Gag and Env or a monoclonal antibody to immunoprecipitate HSV-1 gD. The immunoprecipitates of the HIV-1 proteins and HSV-1 gD from gradient fractions of cells co-transfected with pcDNA3.1(+) and pNL4-3 or gD and pNL4-3 were the same as in Fig. 7. **a** Immunoprecipitation of HIV-1 proteins and HSV-1 gD from cell lysates of cells co-transfected with pcDNA3.1(+) and pNL4-3, a vector expressing Bal Env and pNL4-3, a vector expressing gD and pNL4-3, or a vector expressing gD, Bal Env and pNL4-3. **b** Immunoprecipitation of HIV-1 proteins from supernatants prior to and after ultracentrifugation through a 20% sucrose cushion from cells co-transfected with pcDNA3.1 and pNL4-3, a vector expressing Bal Env and pNL4-3, a vector expressing gD and pNL4-3, or a vector expressing gD and Bal Env and pNL-3. **c** Immunoprecipitation of HSV-1 gD from viral pellet after ultracentrifugation through a 20% sucrose cushion from cells transfected with pcDNA3.1(+) and pNL4-3, a vector expressing gD and pNL4-3, a vector expressing Bal Env and pNL4-3, or a vector expressing gD, Bal Env, and pNL4-3. **d** Immunoprecipitation of HIV-1 proteins from gradient fractions of cells co-transfected with Bal Env and pNL4-3. **e**, **f** Immunoprecipitation of HIV-1 proteins (**e**) and HSV-1 gD (**f**) from gradient fractions of cells co-transfected with a vector expressing gD, Bal Env, and pNL4-3

One interpretation of the above results could be that over-expression of gD out competed the gp120/gp41 for incorporation into particles. To address this potential scenario, we next over-expressed both gD and HIV-1 gp160 to determine if gp120/gp41 would be excluded from maturing virus particles. 293 cells were transfected with vectors expressing HIV-1 Bal gp160, HSV-1 gD, or both gD and HIV-1 Bal gp160 and pNL4-3. Both the gD and HIV-1 Bal gp160 were expressed from the same CMV IE promoter. At 30 h, cells were starved and radiolabeled with ^35^S-methionine/cysteine for 16 h. At 48 h, the virus was collected, pelleted through a sucrose cushion, and analyzed by immunoprecipitation analysis for the presence of HIV-1 p24, gp120/gp41, and gD. In the cells transfected with pcDNA3.1(+) and pNL4-3, gp160/gp120, and p24 were readily detected in the cell lysates and gp120 and p24 in the culture medium (Fig. 8a, b). Cells transfected with the vector expressing Bal Env and pNL4-3 also resulted in the gp160/120, p55, and p24 in cell lysates although the level of gp160/gp120 were higher as expected (Fig. 8a). This was also reflected in the levels of gp120 released in the culture medium (Fig. 8b). Similarly, the co-expression of HSV-1 gD and HIV-1 NL4-3 resulted in the p55/p24 and gp160/gp120 in the cell lysates and gp120 and p24 in the culture medium, although the levels of Env immunoprecipitated were consistently less than the pcDNA3.1(+)/pNL4-3 control (Fig. 8a, b). Finally, expression of gD, HIV-1 Bal Env, and HIV-1 NL4-3 resulted in higher levels of gp160/gp120 in cell lysates and higher levels of gp120 in the culture medium compared to cells co-transfected with gD/pNL4-3 (Fig. 8a, b). The levels of gD in the cell lysates was similar for cells transfected with gD/pNL4-3 or Bal Env/gD/pNL4-3 (Fig. 8a). Next, we loaded the culture medium onto a 20% sucrose cushion and pelleted the virus by ultracentrifugation. Our results indicate that similar levels of gp120 were immunoprecipitated from the virus pellet derived from pcDNA3.1(+)/pNL4-3 and Bal Env/pNL4-3 transfected cells (Fig. 8b). The virus pellets derived from gD/pNL4-3 and gD/Bal Env/pNL4-3 transfected cells revealed a significant reduction of gp120 in the pellets, indicating less Env in the pelleted virus (Fig. 8b). Furthermore, the amount of gD in the virus pellet derived from gD/Bal Env/pNL4-3 transfected cells was much less compared to gD/pNL4-3 transfected cells (Fig. 8c). To determine if the gp120 observed in the virus pellets from gD/pNL4-3 and gD/Bal Env/pNL4-3 was associated with virus, the virus pellets were resuspended and layered onto 20–60% sucrose gradients. These results indicate that while gp120 was associated with virus from Bal Env/pNL4-3 transfected cells, gp120 was not detected in virus from cells co-transfected with gD/Bal Env/pNL4-3 transfected cells after ultracentrifugation through gradients (Fig. 8d, e). These results show that over-expression of both glycoproteins still resulted in the exclusion of the HIV-1 Env from the maturing virus particles, suggesting that competition for incorporation was likely not the mechanism of HIV-1 Env exclusion. We also performed a titration of the vector expressing gD to determine if reduced levels of plasmid would also result in restriction of the release of infectious HIV-1. Our results indicate that a 10-fold reduction in the level of plasmid also resulted in statistically significant restriction in the release of infectious HIV-1 (data not shown). Taken, together, these results indicate that lower levels of gD expression was sufficient for restriction of HIV-1.

### The cytoplasmic domain of gD is not critical for the restriction of HIV-1

**Fig. 9:**
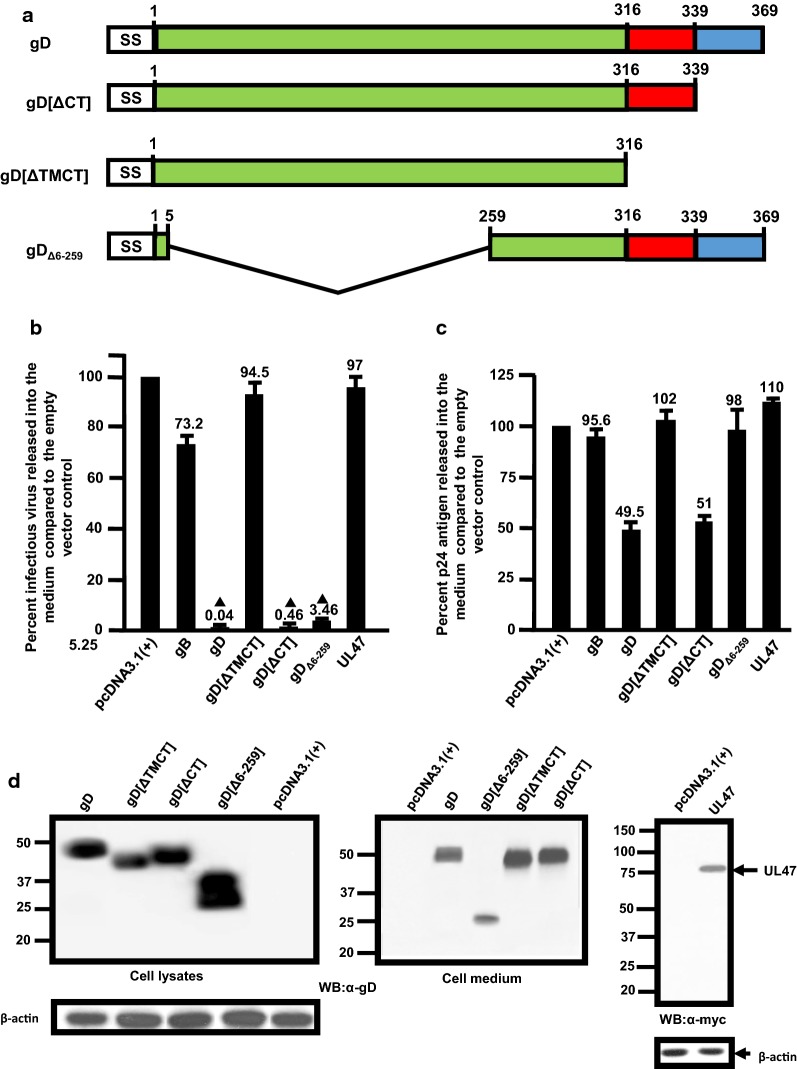
Analysis of gD mutants for the ability to restrict the release of infectious HIV-1. 293 cells were co-transfected with vectors expressing gD, gD[ΔTMCT], gD[ΔCT], and gD _[6−259]_, gB (negative control), or UL47 (negative control) and a plasmid pNL4-3. At 48 h, the culture supernatants were collected and the levels of infectious virus released into the culture supernatants was determined using TZM-bl cell assays and levels of p24 determined using p24 kits. **a** Schematic diagram of the gD mutants analyzed. **b** Results of restriction analysis of gD and different gD mutants. **c** The levels of p24 protein released from cells from the experiments in (**b**). **d** The expression of each gD protein and β-actin (left two panels), gD proteins released into the culture medium (middle panel) and from UL47 and β-actin (right panels) from a representative restriction assay using immunoblots. The experiments were performed at least four times and statistical differences with the pcDNA3.1(+)/HIV-1 control evaluated using a two-tailed Student’s *t*-test, with *p *< 0.01 (filled triangle) considered significant

We analyzed three truncated forms of the HSV-1 gD with the first having the transmembrane and cytoplasmic domains of protein deleted (gD[ΔTM/CT]), a second in which the cytoplasmic domain was deleted (gD[ΔCT]), and a third with a large region of the ectodomain deleted (gD_Δ6−259_) [24; Fig. 9a]. We analyzed the ability of these three gD mutants to restrict HIV-1. 293 cells were transfected with the empty pcDNA3.1(+) vector, one expressing either gD, gD[ΔTM/CT], gD[ΔCT], gD_Δ6−259_, gB, or UL47 and pNL4-3. Cells were incubated for 48 h and the culture medium collected to analyze for infectious virus using TZM-bl assays and for p24 levels using commercially available kits. The results show a high level of restriction of infectious virus release in cells co-transfected with gD (0.04% release compared to control) and gD[ΔCT] (0.46% compared to control) while the restriction of infectious HIV-1 release in the presence of gD_Δ6−259_ was approximately 3.5% of the control (Fig. 9b). Neither gD[ΔTM/CT] nor gB significantly restricted the release of infectious HIV-1 (Fig. 9b). The levels of p24 released into the culture medium revealed that both gD and gD[ΔCT] were approximately 50% while the levels of p24 released from cells co-transfected with gD[ΔTMCT], and gD_Δ6−259_/pNL4-3, gB/pNL4-3, and UL47/NL4-3 were approximately 90–115% of the control (Fig. 9c). Finally, we analyzed the expression of the gD (or gD mutant) expression from the restriction assay using an immunoblot and a monoclonal antibody directed against gD. The results of the immunoblot analysis show the relative size of gD[ΔTM/CT], gD[ΔCT], and gD_Δ6−259_ in relation to the unmodified gD in cell lysates (Fig. 9d). As expected, immunoprecipitation of gD proteins from the culture medium revealed that gD[ΔTM/CT] was efficiently released into the culture medium compared to gD, gD[ΔCT], and gD_Δ6−259_ (Fig. 9d) although the levels of gD_Δ6−259_ in the culture medium were much reduced. Taken together, these results indicate that removal of the C-terminus of gD still reduced the release of infectious virus although the level of restriction was approximately 10-fold less than the unmodified gD and removal of V-like immunoglobulin region of the ectodomain still resulted in significant restriction of HIV-1, although it was approximately 100-fold less than the unmodified gD. Finally, our results indicate that a membrane anchored gD was likely necessary for activity.

### The gD from HSV-2 also significantly restricts HIV-1

**Fig. 10:**
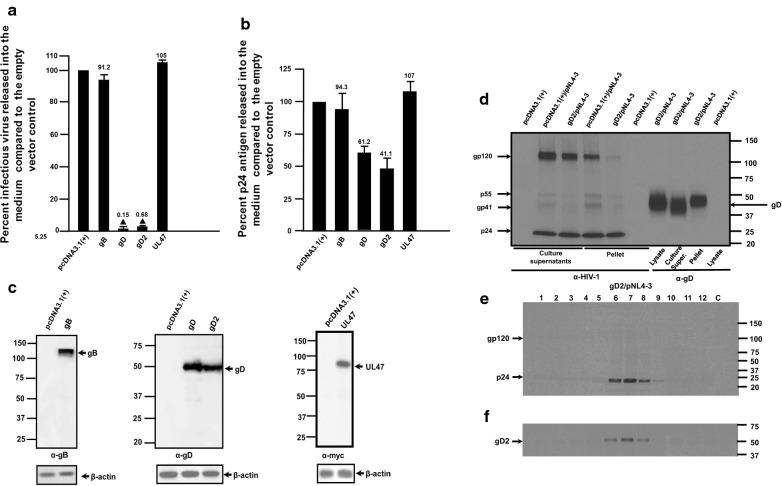
The gD protein from HSV-2 also restricts the release of infectious HIV-1. 293 cells were co-transfected with either the empty pcDNA3.1(+) vector or one expressing gD, HSV-2 gD (gD2), gB, or UL47 and pNL4-3. At 48 h, the culture supernatants were collected. The levels of infectious virus released into the culture supernatants was determined using TZM-bl cell assays and p24 in the culture supernatants determined using p24 antigen capture assays. **a** The level of infectious virus released into the culture medium from cells co-transfected with pcDNA3.1(+), gD, HSV-2 gD (gD2), gB, or UL47 and pNL4-3. **b** The levels of p24 protein produced from cells co-transfected with pcDNA3.1(+), gD, HSV-2 gD (gD2), gB, or UL47 and pNL4-3. **c** The cell lysates from the above restriction assays were analyzed for the presence of gD, HSV-2 gD (gD2), gB, or UL47 using Western blots and appropriate antibodies. **d** 293 cells were transfected with empty vector pcDNA3.1(+), pcDNA3.1(+) and pNL4-3, or the vector expressing gD2 and pNL4-3. At 30 h post-transfection, cultures were radiolabelled as per Fig. 8. At 48 h, the culture medium was harvested, centrifuged at low speed to remove cellular debris and layered onto a 20% sucrose cushion. The virus containing medium was subjected to ultracentrifugation to pellet the virus and HIV-1 proteins and gD2 immunoprecipitated using appropriate antibodies. The samples were analyzed by SDS-PAGE and standard radiographic techniques. **e**, **f** A companion experiment was performed exactly like **d** except the pelleted virus resuspended in DMEM without serum and layered on a discontinuous 20–60% sucrose gradient. The virus was subjected to ultracentrifugation for 20 h (76,000 x g, SW55Ti rotor), 12 fractions were collected, and subjected to immunoprecipitation analysis using anti-HIV-1 antibodies to immunoprecipitated HIV-1 proteins (Gag and Env) and an appropriate monoclonal antibody to immunoprecipitate HSV-1 gD2. The immunoprecipitates were collected on protein-A-Sepharose beads, washed, and boiled in sample reducing buffer. The proteins were separated on 7.5% SDS-PAGE and visualized using standard radiographic techniques. The experiments in **a**–**c** were performed at least four times and statistical differences with the pcDNA3.1(+)/HIV-1 control evaluated using a two-tailed Student’s *t*-test, with *p *< 0.01 (filled triangle) considered significant

In a previous study, the gD from HSV-2 was shown not to be incorporated into HIV-1 particles [25]. An alignment of the gD proteins of HSV-1 (F strain) and HSV-2 (HG52 strain) revealed approximately 85% identity at the amino acid level (data not shown). We next determined if the gD from HSV-2 (gD2) would restrict HIV-1. Restriction analyses revealed that like gD, gD2 also potently restricted the release of infectious HIV-1 while gB did not restrict the release of infectious HIV-1 (Fig. 10a). Cells co-transfected with pNL4-3 and the vector expressing gD2 released similar levels of p24 compared to gD and was expressed at similar levels as gD (Fig. 10b, c). We also examined virus produced in the presence of gD2. 293 cells were co-transfected with the vector expressing gD2 and pNL4-3 or pcDNA3.1(+) and pNL4-3 were radiolabeled at 30 h post-transfection and the virus containing culture medium was harvested at 48 h post-transfection. Immunoprecipitation of HIV-1 proteins from the culture medium revealed similar levels of gp120 and p24 released in cells co-transfected with pcDNA3.1(+) and pNL4-3 or the vector expressing gD2 and pNL4-3 (Fig. 10d). We also layered the culture medium onto a 20% sucrose cushion and pelleted the virus by ultracentrifugation. Our results revealed that both gp120 and p24 were observed in the virus from cells co-transfected with pcDNA3.1(+) and pNL4-3 (Fig. 10d). However, very little gp120 was observed in virus pelleted from cells co-transfected with the vector expressing gD2 and pNL4-3 (Fig. 10d). Additionally, examination of the virus pellets for the presence of gD2 by immunoprecipitation revealed gD2 was present in the virus pellets. Finally, we partially purified the pelleted virus on 20–60% sucrose gradients and examined the fractions for HIV-1 proteins and gD2 by immunoprecipitation. The results reveal that like gD, the presence of gD2 excluded HIV-1 Env from being incorporated into the virus particles while gD2 was detected in the same fractions having p24 (Fig. 10e, f). Taken together, these results indicate that from a mechanistic standpoint, both gD and gD2 excluded HIV-1 Env from particles in a similar manner.

### Immunofluorescence studies show that gD and gB co-localize with Gag-EGFP at the cell plasma membrane

**Fig. 11:**
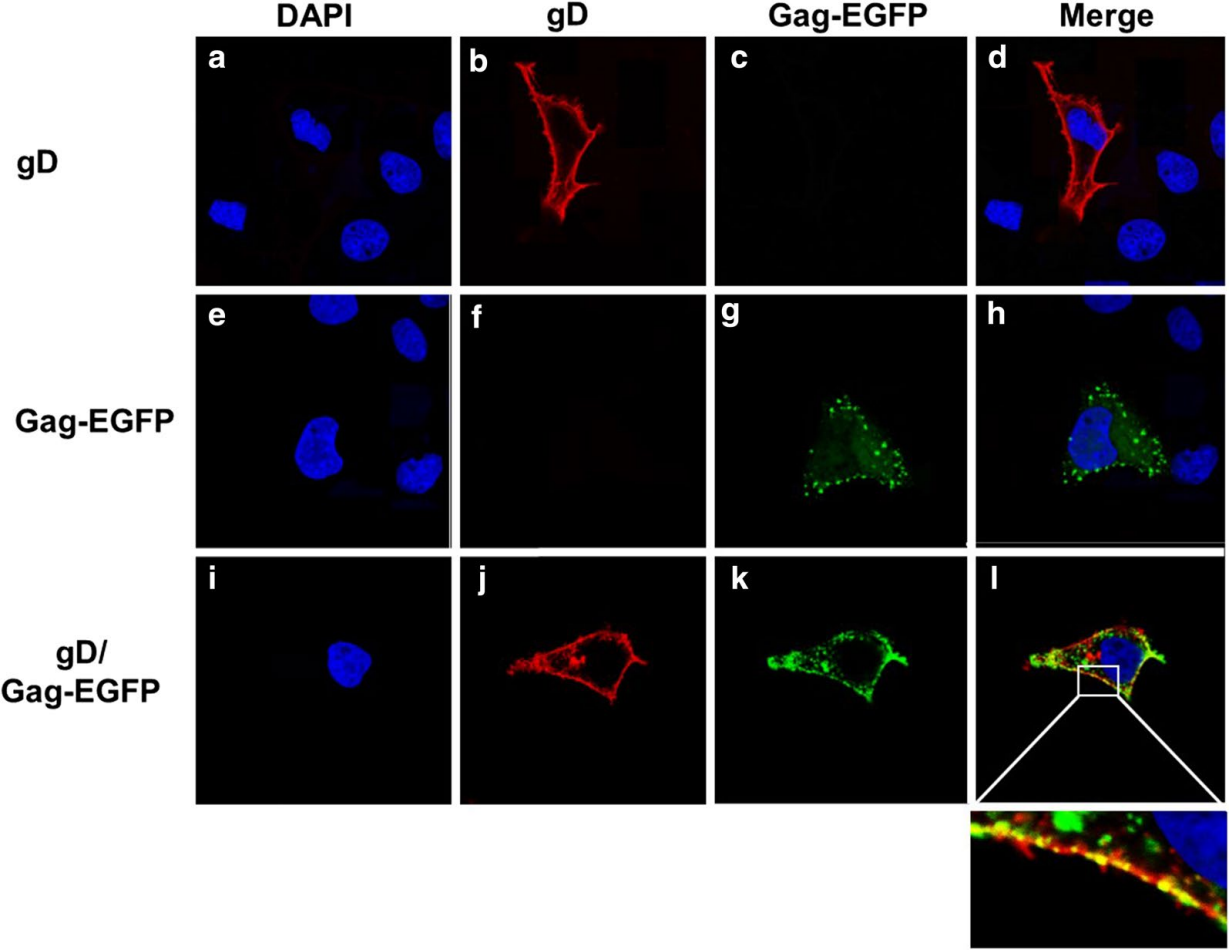
The HSV-1 gD co-localizes with Gag-EGFP. 293 cells were transfected with either the vector expressing gD, Gag-EGFP, or co-transfected with gD and Gag-EGFP. At 24 h, cells on cover slips were washed, fixed, permeabilized and blocked as described in the Materials and Methods section. The cover slips were reacted with a mouse monoclonal antibody against gD and an appropriate secondary antibody and counterstained with DAPI (1 μg/ml) for 5 min. The cover slips were mounted and examined using a Leica TCS SPE confocal microscope using a 63X objective with a 2X digital zoom using the Leica Application Suite X (LAS X, LASX) software package. A 405 nm filter used to visualize the DAPI, 488 nm filter was used to visualize the Gag-EGFP, and a 594 nm filter to visualize gD staining. **a**–**d** Cells transfected with vector expressing gD. **e**–**h** Cells transfected with vector expressing Gag-EGFP. **i**–**l** Cells co-transfected with vectors expressing gD and Gag-GFP. **a**, **e**, **i** Visualization of DAPI staining. **b**, **f**, **j** Visualization of gD staining. **c**, **g**, **k** Visualization of Gag-EGFP. **d**, **h**, **l** Merge of the three panels to the left

**Fig. 12:**
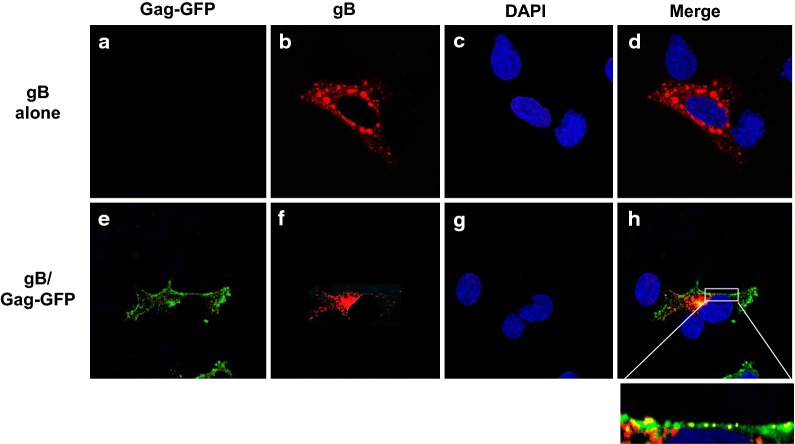
The HSV-1 gB co-localizes with Gag-GFP at the cell surface. COS-7 cells were transfected with either the vector expressing gB or co-transfected with gB and Gag-EGFP. At 24 h, cells on cover slips were washed, fixed, permeabilized and blocked as described above. The cover slips were reacted with a mouse monoclonal antibody against HSV-1 gB and an appropriate secondary antibody and counterstained with DAPI (1 μg/ml) for 5 min as described in the Materials and Methods section. The cover slips were mounted and examined using a Leica TCS SPE confocal microscope using a 63 × objective with a 2 × digital zoom using the Leica Application Suite X (LAS X, LASX) software package. A 405 nm filter used to visualize the DAPI, 488 nm filter was used to visualize the Gag-GFP, and a 594 nm filter to visualize gB staining. **a**–**d** Cells transfected with vector expressing gB. **e**–**h** Cells co-transfected with vectors expressing gB and Gag-GFP. **a**, **e** Visualization of Gag-EGFP. **b**, **f** Visualization of gB staining. **c**, **g** Visualization of DAPI staining. **d**, **h** Merge of the three panels to the left. The inset below Fig. 11h is a region of the cell plasma membrane showing co-localization of Gag-EGFP and gB. The results shown are representative of examining 50 transfected cells

We next examined whether the Gag precursor protein would co-localize with gD and gB. Transfection of COS-7 cells with the vector expressing gD followed by intracellular staining using an anti-gD antibody revealed that gD was easily detectable at the cell plasma membrane (Fig. 11a–d). Transfection of COS-7 cells with a with codon optimized Gag-EGFP revealed a punctate staining generally at the cell surface (Fig. 11e–h), which is like a previously reported study [26]. We next co-transfected cells with vectors expressing gD and Gag-EGFP (Fig. 11i–l). We detected co-localization of gD with Gag-EGFP as yellow punctate structures that co-localized with Gag-EGFP at the cell plasma membrane (Fig. 11l; see inset). Unlike the gD, HSV-1 gB has several sorting motifs (two tyrosine based motifs and dileucine motif) in its cytoplasmic domain and is known to be quickly recycled from the cell surface [27, 28]. Further, gB has previously been shown to accumulate in larger vesicles and it has been suggested that gB expression may induce structural changes by formation of enlarged vesicles [29]. Transfection of cells with the vector expressing gB revealed that very little gB was observed on the cell surface with most of the protein detectable at intracellular compartments (Fig. 12a–d). Like a previous study, we observed gB associated with large intracellular vesicles [29]. Co-transfection of COS-7 cells with vectors expressing gB and Gag-EGFP resulted in both gB and Gag-EGFP co-localization at intracellular compartments of the cell but some co-localization of the two proteins was observed at the cell surface (Fig. 12e–h). It should be noted fewer large vesicles staining for gB were observed in co-transfected cells.

### Transport of HIV-1 Env to the cell surface is not significantly different in the presence of gD and gB

**Fig. 13:**
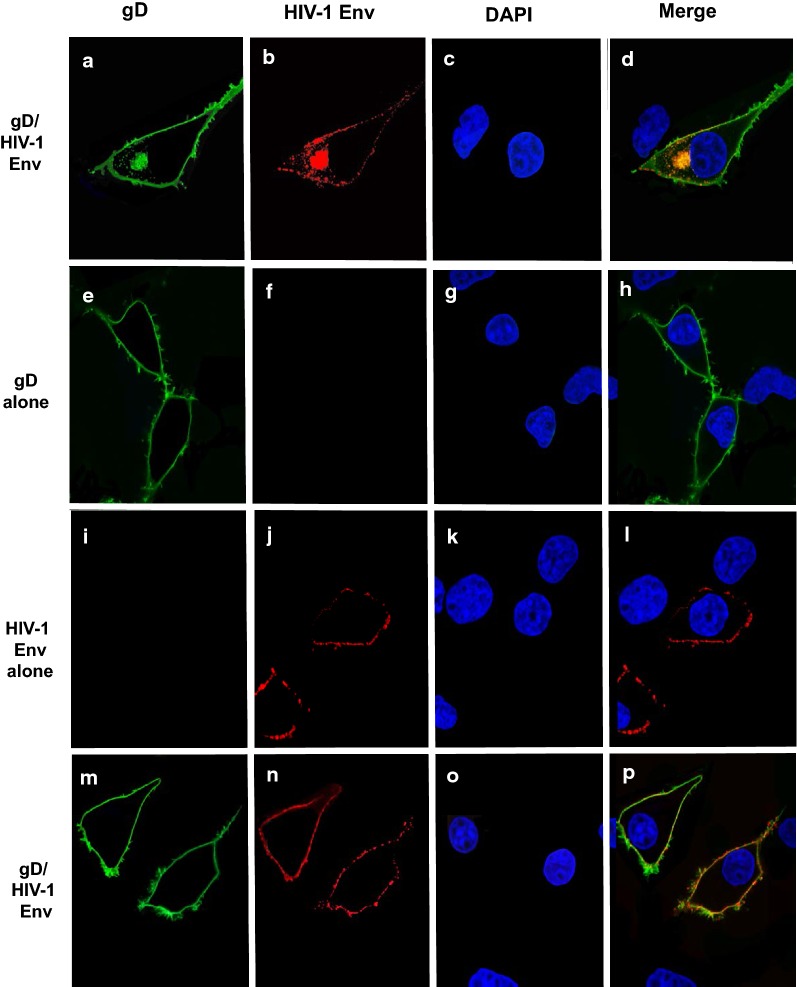
Intracellular and surface expression of HIV-1 Env in the presence of gD. We examined the intracellular and surface expression of gD, HIV-1 Env, or both using COS-7 cells. For intracellular expression, cells grown on cover slips were transfected with vectors expressing gD and HIV-1. At 24 h, cells were fixed, permeabilized, and reacted with a primary antibody against HIV-1 Env and gD for 16 h at 4C. The cells washed and reacted with appropriate secondary antibodies. Cells were counterstained with DAPI. For surface staining, cells on cover slips were transfected with vectors expressing gD, HIV-1 Env or both. At 24 h, cells were washed reacted with primary antibodies against gD and/or HIV-1 Env for 1 h at 4C, cells washed and fixed. The cells were then reacted with appropriate secondary antibodies and counterstained with DAPI. The cells were washed and the cover slips were mounted. The cells on coverslips were examined using a Leica TCS SPE confocal microscope using a 63 × objective with a 2 × digital zoom using the Leica Application Suite X (LAS X, LASX) software package. A 405 nm filter used to visualize the DAPI, 488 nm filter was used to visualize the gD, and a 594 nm filter to visualize HIV-1 Env staining. **a**–**d** Cells co-transfected with vectors expressing gD and HIV-1 Env that were fixed prior to staining. **e**–**h** Cells transfected with a vector expressing gD and reacted with the primary antibody against gD at 4C prior to fixation. **i**–**l** Cells transfected with vectors expressing HIV-1 Env and reacted with the primary antibody against HIV-1 Env at 4C prior to fixation. **m**–**p** Cells co-transfected with vectors expressing HIV-1 Env and gD, reacted with the primary antibodies against HIV-1 Env and gD at 4C prior to fixation. The results shown are representative of examining 50 transfected cells. **a**, **e**, **i**, **m** Visualization of gD staining. **b**, **f**, **j**, **n** Visualization of HIV-1 Env staining. **c**, **g**, **k**, **o** Visualization of DAPI staining. **d**, **h**, **l**, **p** Merge of the three panels to the left

**Fig. 14:**
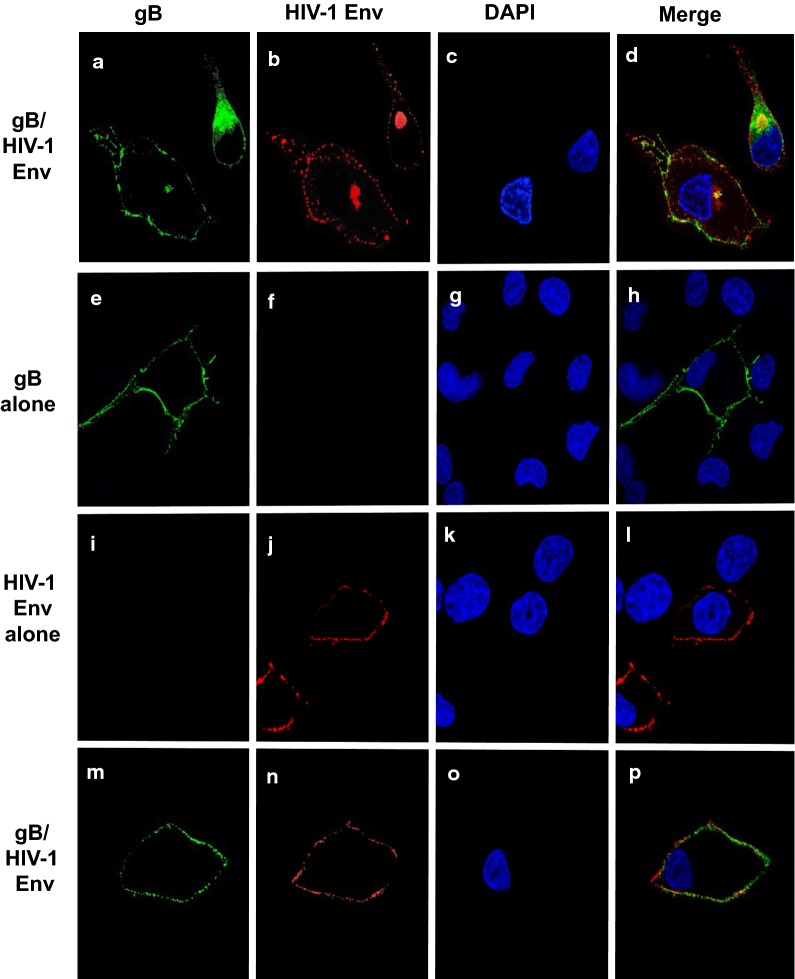
Intracellular and surface expression of HIV-1 Env in the presence of gB. We examined the intracellular and surface expression of gB, HIV-1 Env, or both using COS-7 cells. For intracellular expression, cells grown on cover slips were transfected with vectors expressing gB and HIV-1. At 24 h, cells were fixed, permeabilized, reacted with a primary antibody against HIV-1 Env and gB for 16 h at 4C. The cells washed and reacted with appropriate secondary antibodies. Cells were counterstained with DAPI. For surface staining, cells on cover slips were transfected with vectors expressing gB, HIV-1 Env or both. At 24 h, cells were washed reacted with primary antibodies against gB and/or HIV-1 Env for 1 h at 4C, cells washed and fixed. The cells were then reacted with appropriate secondary antibodies and counterstained with DAPI. The cells were washed and the cover slips were mounted. The cells on coverslips were examined using a Leica TCS SPE confocal microscope using a 63 × objective with a 2 × digital zoom using the Leica Application Suite X (LAS X, LASX) software package. A 405 nm filter used to visualize the DAPI, 488 nm filter was used to visualize the gB, and a 594 nm filter to visualize HIV-1 Env staining. **a**–**d** Cells co-transfected with vectors expressing gB and HIV-1 Env that were fixed prior to staining. **e**–**h** Cells transfected with a vector expressing gB and reacted with the primary antibody against gB at 4C prior to fixation. **i**–**l** Cells transfected with vectors expressing HIV-1 Env and reacted with the primary antibody against HIV-1 Env at 4C prior to fixation. **m**–**p** Cells transfected with vectors expressing HIV-1 Env and gB, reacted with the primary antibodies against HIV-1 Env and gB at 4C prior to fixation. The results shown are representative of examining 50 transfected cells. **a**, **e**, **i**, **m** Visualization of gB staining. **b**, **f**, **j**, **n** Visualization of HIV-1 Env staining. **c**, **g**, **k**, **o** Visualization of DAPI staining. **d**, **h**, **l**, **p** Merge of the three panels to the left

We next analyzed the cell surface expression of HIV-1 Env in the presence of gD or gB. COS-7 cells were transfected with vectors expressing HIV-1 Env alone, gD alone, gB alone, HIV-1 Env and gD, or HIV-1 Env and gB. Two sets of experiments were performed. In the first experiment, cells were co-transfected with vectors expressing gD and HIV-1, cells were fixed at 24 h post-transfection and stained with appropriate primary and secondary antibodies. The results indicate that gD appeared to be expressed uniformly on the cell surface while HIV-1 Env was expressed at intracellular compartments and at the cell surface in a punctate staining pattern (Fig. 13a–d). We also analyzed the surface expression of each protein or together. Here cells were transfected and at 24 h, cells washed and reacted with primary antibodies at 4 C for 1 h. After washing cells, they were fixed and then reacted with the secondary antibodies. In this scenario, expression of gD alone revealed a uniform cell surface staining pattern while HIV-1 Env showed a punctate staining pattern (Fig. 13e–l). This was also observed when cells were co-transfected with vectors expressing gD and HIV-1 Env (Fig. 13m–p). We also examined gB/HIV-1 Env expression using these two methods. Co-transfection of cells with vectors expressing gB and HIV-1 Env followed by fixation at 24 h revealed that the majority of gB was observed at intracellular compartments with some expression at the cell surface although we did observe some cells where gB expression was confined to intracellular compartments. Similarly, the HIV-1 Env was observed both at intracellular compartments and at the cell surface (Fig. 14a–d). Examination of cell surface expression of gB alone, HIV-1 Env alone or together revealed a similar expression patterns (Fig. 14e–p). Taken together, it appears that expression of gD or gB did not significantly alter the intracellular trafficking to the cell surface.

### Cell lines stably expressing gD also restrict HIV-1 production

**Fig. 15:**
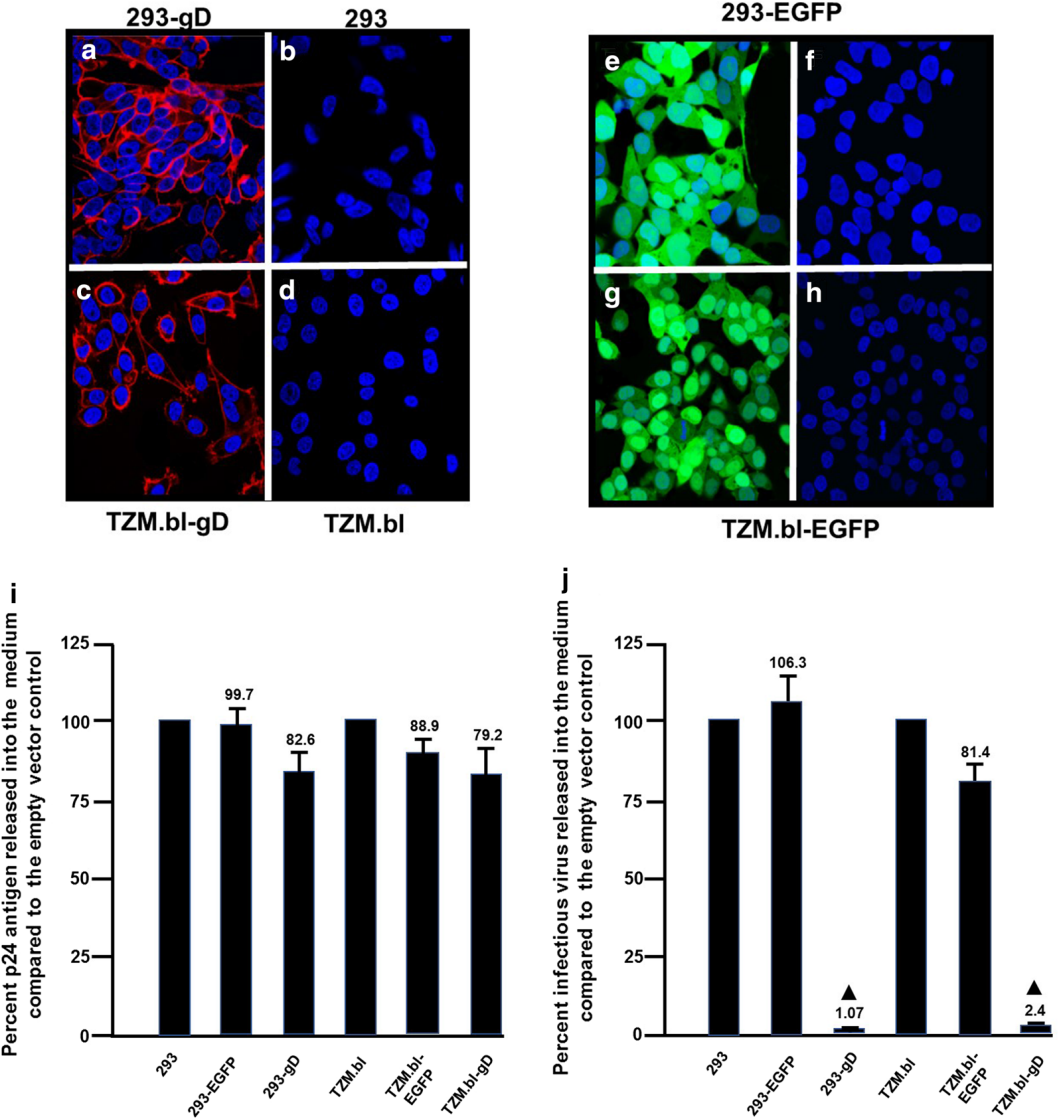
Cell lines expressing gD also restrict HIV-1. 293 and TZM-bl cell lines were transduced with lentivirus vectors expressing HSV-1 gD as described in the Materials and Methods section. Transduced cells were selected using puromycin and gD expressing cell lines were stained for gD and sorted. The number of cells expressing gD was greater than 98%. Cells lines in 6 well plates were inoculated with HIV-1 (NL4-3, complete genome) pseudotyped with the VSV G-protein at an M.O.I of 0.5. At 48 h post-inoculation, the medium was collected and the levels of p24 determined with commercially available p24 ELISA kits and infectious virus released determined using TZM-bl assays. **a**–**d** Parental 293 and TZM-bl cell lines and those expressing HSV-1 gD (293-gD, TZM-bl-gD) were fixed and immunostained for gD and examined by confocal microscopy. **e**–**h** Parental 293 and TZM-bl cell lines and those expressing EGFP (293-EGFP, TZM-bl-EGFP) were examined for the expression of EGFP by confocal microscopy using a 488 nm filter. **i** The levels of the p24 released from cells inoculated with VSV-G pseudotyped HIV-1. **j** The levels of infectious virus released from cells inoculated with VSV-G pseudotyped HIV-1. The experiments were performed at least three times and statistical differences with the 293 or TZM-bl/HIV-1 controls were evaluated using a two-tailed Student’s *t*-test, with *p *< 0.01 (filled triangle) considered significant. The numbers above the bars represent the percentage of p24 or infectious virus compared to the control

Based on the above results with gD, we constructed 293 and TZM-bl cell lines expressing the full length gD using lentiviral vectors containing a puromycin selectable marker. Following transduction, cells were selected with puromycin according to the manufacturer’s suggestions. The surviving cells were stained for the presence of gD and flow sorted for cells expressing gD. Evaluation of the sorted cells revealed that greater than 98% of the cells expressed gD (Fig. 15a, c) while the parental cell lines showed no staining for gD (Fig. 14b, d). We also generated 293 and TZM-bl cell lines with the same lentivirus vector expressing EGFP using the same lentivirus vector (293-EGFP, TZM-bl-EGFP) showed no staining for gD (Fig. 15e–h). Both sets of cell lines were inoculated with infectious NL4-3 pseudotyped with VSV-G virus at a multiplicity of infection (M.O.I.) of 0.5. At 48 h, the culture medium was collected and the levels of p24 released determined by ELISA assays and the levels of infectious virus determined using TZM-bl assays (Fig. 15i, j). The results show that the 293[gD] and TZM-bl[gD] cells potently restricted the release of infectious virus at levels of approximately 1% and 2.5% of the control cells, respectively. These results correlate well with our transient transfection results and provide additional evidence that gD can restrict the release of infectious HIV-1.

## Discussion

Previously, we showed that several HSV-1 proteins could inhibit the production of infectious HIV-1 from co-transfected cells and that the HSV-1 gM protein interfered with the processing and transport of the HIV-1 Env precursor, which resulted in its degradation and prevented its incorporation into maturing virus particles [17]. In this study, we have assessed whether four HSV-1 proteins (gH, gL, gD, and gB), previously shown to be responsible for HSV-1 entry into cells, would disrupt the production of infectious HIV-1. Our results indicate that of these four glycoproteins, the HSV-1 gD and the gH/gL complex restricted the production of infectious HIV-1 while gB had no significant effect. Of these glycoproteins, gD most potently restricted HIV-1 production. Our studies indicate that HSV-1 gD is incorporated into HIV-1 particles while apparently excluding the incorporation of HIV-1 gp120/gp41.

In a previous study, investigators showed that transfection of HSV-2 gD (gD2) into HIV-1 infected cells did not result in the incorporation of gD2 into the viral membrane [25]. In this study, investigators used a viral capture assay using antibodies directed against gD and quantified virus by analysis of viral RNA content [25]. We also analyzed the ability of gD2 to restrict the release of infectious HIV-1. Our results indicate that not only did gD2 restrict the release of infectious HIV-1 but it was also incorporated into virus particles as partial purification of virus through sucrose density gradients. This suggests that gD and gD2 most likely acted through a similar mechanism to restrict HIV-1. One potential mechanism for the specific incorporation of gD or gD2 into HIV-1 particles and exclusion of the HIV-1 gp120/gp41 would be that gD out-competed HIV-1 Env for incorporation due to over-expression. Several pieces of data would argue against this scenario. First, we performed experiments in which we expressed HIV-1 Env from the same CMV promoter that gD was expressed. Our results were similar. Second, if it was a simple out-competition, one would expect that expression of gB (also a type 1 membrane protein) in the presence of NL4-3 would lead to incorporation into virus while excluding HIV-1 Env. However, expression of gB had no significant effect on the production of infectious HIV-1 production and was incorporated into the HIV-1 particles along with the gp120/gp41. Finally, we titrated the gD plasmid and showed that there was a similar level of restriction at 1/10 the amount of plasmid. Taken together, these results indicate that gD-mediated exclusion of HIV-1 Env was specific.

The X-ray structure of HSV-1 gD ectodomain was previously solved [30, 31]. The mature form of the gD of HSV-1 is a 369-residue glycoprotein with an N-terminal ectodomain of 316 residues and three N-linked oligosaccharide attachment sites [32]. It is a type I membrane protein with a single transmembrane domain and cytoplasmic tail of 30 amino acids. The protein has six cysteines that form three disulfide bonds (Cys 66-Cys 189, Cys 106-Cys 202, and Cys 118-Cys 127) [33]. The N-terminus of gD is flexible and when extended folds into a hairpin structure that binds herpes virus entry mediator (HVEM). The core domain (amino acids 28–260) consists of a V-like immunoglobulin (Ig) fold. Truncation of the gD ectodomain to residues 285 (gD285) or 275 (gD275) both block infection and bind to receptors in vitro 100 times better than a longer form (gD306) [12, 34–36]. The C-terminal region (known as the profusion domain) of the gD ectodomain (residues 260–316) is characterized by the presence of 11 proline residues (11/56 or ~ 20% of the residues) and data suggests it is an unstructured region. The C-terminus also plays an important functional role in HSV entry as virions carrying forms of gD with insertions/deletions between residues 275 and 300, or a chimera containing amino acids 1–260 of gD linked to domains of CD8 were impaired in cell entry [37–39]. Our deletion mutant studies of HSV-1 gD showed that removal of the cytoplasmic tail of gD did not affect HIV-1 restriction while removal of V-like domain from the ectodomain (amino acids 6–259) resulted in an approximately 100-fold reduction in restriction, although this level of restriction was found to be statistically significant. Finally, removal of both the transmembrane (TM) and cytoplasmic domains eliminated restriction properties of gD. It is possible that the lack of TM region may affect the localization and trafficking of the gD, which consequently prevents it from restricting Env incorporation, rather than a direct effect of the TM region. It will be of interest to determine if the pro-fusion domain (amino acids 260–316) has the determinants for gD that restrict the release of infectious HIV-1 and whether substitution of the TM domain of gD (amino acids 317–339) with the TM of another, non-restrictive glycoprotein such as gB would also restrict infectious HIV-1 release.

The mechanism through which gD inhibited the release of infectious HIV-1 from cells was not due to the synthesis of gp160, cleavage into the gp120/gp41 heterodimer, or transport of gp120/gp41. However, our pulse-chase experiments did show that there was slightly less gp160 synthesized and cleaved gp120. These results suggest that minor (slightly less Env synthesized and processed) and major (exclusion of HIV-1 Env) mechanisms may be involved. Our results indicate that the mechanism of gD restriction was likely due to the exclusion of gp120/gp41 from maturing viruses, which would explain the very low infectious titers of virus released from cells. Further, the mechanism of gD-mediated gp120/gp41 exclusion from virus appears to be specific as the HSV-1 gB, while also released from cells, had neither a significant effect on the infectivity of the released HIV-1 nor the incorporation of HIV gp120/gp41 into infectious virus particles. Thus, a salient question is, “Why does the presence gD inhibit the incorporation of gp120/gp41 into virus particles?” One possible explanation for the incorporation of gD comes from transport of these glycoproteins during HSV-1 replication. The localization of cellular (and viral) membrane proteins is usually mediated through discrete targeting motifs present in cytoplasmic domains [40]. These motifs mediate their inclusion into transport vesicles via direct or indirect interactions with the vesicle forming coat machinery. Such targeting motifs include the tyrosine-based motifs (YXXΦ, where X indicates any amino acid and Φ indicates an amino acid with a bulky hydrophobic side chain) and dileucine motifs (D/E]XXXL[L/I]), which interact with clathrin adaptor complexes. Of the four HSV-1 glycoproteins examined here, the gB cytoplasmic domain has two canonical YxxΦ motif and one dileucine motif, which mediates its localization to intracellular assembly sites. On the other hand, the gD and gH/gL proteins have no tyrosine-based or dileucine-based internalization signals in their cytoplasmic tails but rather rely on other viral glycoproteins for targeting them to the sites of assembly [41, 42]. Both gM and gK/UL20 were shown to mediate internalization of gD and gH/gL [40, 42]. Thus, ectopic expression of gD in the absence of other HSV-1 proteins results in gD transport to the cell surface but slower internalization. Thus, it is possible that the presence of gD at the cell surface for extended periods of time could increase the likelihood of its incorporation into virus. A previous study with another herpesvirus, pseudorabies virus (PRV), showed that its gD was also transported to the cell surface but was not efficiently endocytosed [43]. These investigators showed that a chimeric protein consisting of the ectodomain and transmembrane domain of gD fused to the cytoplasmic domain of gB resulted in the endocytosis of the chimeric protein [43]. It will be of interest to determine if the introduction of an internalization signal into the cytoplasmic domain of gD results in enhanced endocytosis and decreased ability to restrict the release of infectious HIV-1 and incorporation of HIV-1 Env into maturing viral particles. Another potential factor involved comes from the observations that HSV-1 (and other herpesviruses) are capable of remodeling host cell membranes for virus egress [44]. Ectopic gD expression is known to induce microvillus tubular structures at the plasma membrane, which have been mapped to a cluster of arginines at the membrane proximal region of the cytoplasmic domain [45]. It will also be of interest to determine if removal of these arginines affects the incorporation of gD or HIV-1 Env.

Another question raised by this study is, “Why is gD preferentially incorporated into particles over gp120/gp41?” Four models have been proposed for the incorporation of the HIV-1 Env into virus particles [46–48]. In the passive model, membrane proteins are transported to the cell plasma membrane and randomly present at sites of virus assembly. In a second model, there is co-targeting of Gag and Env to common sites on the plasma membrane, which leads to an increase in the amount of Env packaged into particles. This likely occurs in membrane domains such as lipid rafts [49–54]. In the third model, the Gag precursor protein directly interacts with the gp120/gp41, likely through interactions with the Gag precursor and the cytoplasmic domain of gp41 [55–61]. Finally, a fourth model proposes an indirect interaction between Gag and Env that is mediated by a cellular adaptor protein [62–64]. The finding that gD was preferentially incorporated over gp120/gp41 would argue against the passive incorporation model. Our immunofluorescence studies indicated that both gD and Gag-EGFP partially co-localized at the cell surface as was  gB and Gag-EGFP. Whether the number of gB molecules incorporated is less than gD is currently under investigation.  While we have no data as to whether gD and the Gag precursor directly interact, the data with the gD[ΔCT] suggests that this may be unlikely although our immunofluorescence studies showed co-localization of Gag-EGFP and gD. Finally, the data from our studies is insufficient to argue for or against the indirect interaction model.

The results presented describe a well-studied HSV-1 protein that prevents the incorporation of gp120/gp41 into HIV-1 particles leading to the restricted release of infectious HIV-1. It is possible that further studies on gD could provide insight into structural domains important in restricting HIV-1 gp120/gp41 incorporation and as a tool to further understand the role of viral HIV-1 glycoprotein trafficking and incorporation. Finally, we have shown that cell lines could be established that significantly reduce the release of infectious virus, which could point to potentially new antiviral strategies.

## Methods

### Viruses, cells, and antibodies

293 cells were used for transfection of vectors expressing the HSV-1 proteins. The TZM-bl cell line, which was used as an indicator to measure virus infectivity, was obtained from the NIH AIDS Reagent Branch [20–23]. Both cell lines were maintained in Dulbecco’s minimal essential medium (DMEM) with 10% fetal bovine serum (R10FBS), 10 mM Hepes buffer, pH 7.3, 100 U/ml penicillin, 100 μg/ml streptomycin and 5 μg/ml gentamicin. A plasmid expressing the Rev and gp160 from HIV-1 Env (HIV-1 strain Bal; pBal.26) and a plasmid with the entire HIV-1 NL4-3 genome (pNL4-3) were obtained from the NIH AIDS Reagent Branch. The HSV-1 gB and gH were kindly provided by Dr. Richard Longnecker (Northwestern University). A plasmid expressing the HSV-1 gL (myc-DDK-tagged at C-terminus) was obtained from Origene (gL: catalog number, VC100788; CD8: catalog number RC206608). Expression of HSV-1 proteins was confirmed by transfection with the Turbofect transfection reagent (ThermoFisher) and Western blot analysis using appropriate antibodies. For gD and gB, expression was confirmed using mouse monoclonal antibodies directed against each protein (gB: Thermo-Fisher, #MA1-19265, antibody T111; gD: Santa Cruz Biotechnology, #SC-21719, antibody DL6). For gH and gL, expression was confirmed using a mouse monoclonal antibody directed against gH (Abcam, #ab110227) and an anti-myc antibody (Santa Cruz; #sc9E10).

### Analysis of infectious virus production in the presence of HSV-1 proteins

To analyze the virus restriction properties of the HSV-1 proteins, 293 cells were transfected with either empty pcDNA3.1(+) vector or a vector expressing one of the HSV-1 proteins and pNL4-3. At 48 h post-transfection, the culture medium was collected, clarified by low speed centrifugation and the supernatant analyzed for p24 using antigen capture assays (Zeptometrix: HIV-1 p24 ELISA). Levels of infectious virus in the culture supernatants were titrated on TZM-bl cells [20, 21]. TZM-bl cells have an integrated copy of a retrovirus vector expressing β-galactosidase under the control of HIV-1 LTR. If TZM-bl cells are infected with HIV-1, Tat protein expression will result in the expression of the β-galactosidase and infected cells visualized by fixing and staining with X-gal. All assays were performed at least four times and analyzed for statistical significance using two-tiered Student’s *t* test with cells co-transfected with the empty pcDNA3.1(+) and pNL4-3 set at 100% infectivity.

### Biosynthesis and processing of viral proteins in the presence of HSV-1 proteins

The biosynthesis and processing of viral proteins were examined in the presence of gB, gD, gH, and gL. 293 cells were co-transfected with empty pcDNA3.1(+) or the vector expressing gB, gD, gH, or gL-myc and pNL4-3. At 30 h, the cells were washed and incubated in DMEM without methionine/cysteine for 2 h. The cells were washed and radiolabeled in DMEM containing 500 μCi ^35^S-Translabel (methionine and cysteine, MP Biomedicals) for 16 h. The culture medium was collected, subjected to low speed centrifugation, and made 1X with respect to RIPA (1X RIPA: 50 mM Tris–HCl, pH 7.5; 50 mM NaCl; 0.5% deoxycholate; 0.2% SDS; 10 mM EDTA). Cell lysates were prepared by lysis in RIPA buffer for 10 min on ice. The lysates were centrifuged in a microcentrifuge at 14,000 rpm for 10 min and the supernatants transferred to fresh tubes. HIV-1 proteins were immunoprecipitated with a cocktail of antibodies consisting of: (a) a monkey anti-SHIV antibody (a pooled serum from several pig-tailed macaques inoculated with a non-pathogenic SHIV that was harvested at the time of euthanasia, which recognizes the gp160/gp120 and gp41 proteins of HIV-1); (b) a monoclonal antibody against p24 (#6457 and 6458 from the NIH AIDS Reagent Program) and (c) polyclonal antibodies against the HIV-1 Env (#191, 51, and 189 from the NIH AIDS Reagent Program). This cocktail of antibodies is referred to in the figures as anti-HIV antibodies. The immunoprecipitates were collected by incubation with protein A-Sepharose beads at 4 °C. The beads were washed three times with RIPA buffer, and the samples resuspended in sample reducing buffer. The samples were boiled, proteins separated by SDS-PAGE (7.5% gel), and proteins visualized using standard radiographic techniques.

### Gradient analysis of virus

We analyzed the incorporation of gp120/gp41 and HSV-1 gD into virus particles. 293 cells in 35 mm dishes were co-transfected with either empty pcDNA3.1(+) or one expressing gD or gB and pNL4-3. At 30 h post transfection cells were washed, starved with methionine/cysteine for 2 h and radiolabeled with 500 μCi of ^35^S-methionine/cysteine for 16 h. Culture media was harvested and centrifuged at 800×*g* for 10 min. The supernatant was then pelleted through a 20% sucrose cushion at 110,000×*g* in an SW55Ti for 2 h. The pellet was resuspended in 200 μl of serum free media (DMEM). The resuspend pellet was loaded into a 20–60% discontinuous sucrose gradient and subjected to ultracentrifugation at 76,000×*g* for 20 h in SW55Ti. At this point the fractions were collected and immunoprecipitated for p24, HIV-1 Env, HSV-1 gD and gB.

### Construction of gD mutants

The gD gene in the pcDNA3.1(+) vector was used for the construction of the gD mutants lacking either the transmembrane domain and cytoplasmic domain (gD[ΔTMCT]) or lacking the cytoplasmic domain (gD[ΔCT]). The introductions of mutations were accomplished using a QuikChange II site-directed mutagenesis kit (Agilent) according to the manufacturer’s protocol. All plasmid inserts were sequenced to ensure the validity of the mutations and that no other mutations were introduced during the mutagenesis or cloning processes. Expression of the mutant proteins were confirmed using immunoblots and a mouse monoclonal antibody directed against gD/gD mutants.

### Cellular localization studies of gD and gB in the presence of Gag-GFP and HIV-1 Env

We analyzed the intracellular localization of Gag-EGFP, gD or gB alone and Gag-EGFP/gD or Gag-EGFP/gB together. COS-7 cells were transfected with either empty pcDNA3.1(+) vector or one expressing the HSV-1 gD or gB. Additionally, cells were co-transfected with a vector expressing gD or gB and a vector expressing Gag-EGFP. At 24 h, cells on cover slips were washed, fixed, permeabilized and blocked. The cells on cover slips were reacted with a mouse monoclonal antibody against HSV-1 gD (Santa Cruz, DL6; sc-21719) or against gB (Thermo-Fisher, #MA1-19265, antibody T111) overnight at 4C. The cover slips were washed and then reacted with a secondary Alexa Fluor 594 rabbit anti-mouse IgG (Abcam 150128) for 2 h and counter stained with DAPI (1 μg/ml) for 5 min. The cover slips were examined using a Leica TCS SPE confocal microscope using a 63′ objective with a 2X digital zoom using the Leica Application Suite X (LAS X) software package. A 405 nm filter was used to visualize the DAPI staining, a 488 nm filter to visualize Gag-EGFP and a 594 nm filter to visualize Alexa Fluor 594.

We also examined the expression of HIV-1 Env in the presence of gD or gB. Two types of experiments were performed. In the first set of experiments, COS-7 cells were either transfected with vectors expressing Bal Env (pBal.26) alone, gD alone or gB alone or co-transfected with vectors expressing pBal.26 and gD, or pBal.26 and gB. At 24 h post-transfection, the cells were fixed, permeabilized and reacted with primary antibodies (HIV-1 Env: goat anti-Env; gD: mouse monoclonal antibody DL6; gB: mouse monoclonal antibody T111) and appropriate secondary antibodies (HIV-1 Env: donkey anti-goat Alexa 594 [Abcam: ab150136]); gD and gB: rabbit anti-mouse Alexa 488 [Invitrogen A11059]). The cells were counterstained with DAPI (1 μg/ml) for 5 min. In the second set of experiments, we examined the surface expression of HIV-1 Env, gD, and gB. COS-7 cells were transfected as above and at 24 h, live cells were washed with PBS, and reacted with primary antibodies at 4C for 1 h to prevent internalization of antigen/antibody complexes. After washing with cold PBS, cells were fixed in 4% paraformaldehyde for 30 min. and reacted with the secondary antibodies and then counterstained with DAPI as above. Cells on cover slips were mounted and examined as described above. For all immunofluorescence studies, at least 50 cells were examined and micrographs shown are representative of each staining procedure.

### Construction of cell lines expressing HSV-1 gD and infectivity assays

We constructed a cell line that expressed the gD protein using lentiviral vectors. The pLentiCMV GFP Puro (658-5) vector (a gift from Eric Campeau and Paul Kaufman; Addgene plasmid # 17448) was digested with BamHI and Sal I to release the gene encoding EGFP. The vector was treated with Klenow DNA polymerase to generate blunt ends, isolated and the gene for the HSV-1 gD (also treated with Klenow DNA polymerase) was ligated into the vector using standard subcloning techniques. A subclone was isolated (pLenti-gD Puro) and the gene sequenced to ensure proper orientation of the gene and that no gD sequences were deleted or mutated. The pLenti-gD Puro vector was packaged in 293 cells transfected with pLenti-gD Puro, pCMV delta R8.2 (a gift from Didier Trono; Addgene plasmid #12263) and pVSV-G. As a control, the pLenti CMV EGFP Puro (658-5) was also packaged. The culture medium was harvested at 48 h and used to transduce TZM-bl cells and 293 cells in the presence of polybrene (8 μg/ml) at a low M.O.I. (< 0.1). At 24 h post-transduction, cells were washed with PBS and incubated in medium containing puromycin (1 μg/ml for TZM-bl cells; 2 μg/ml for 293 cells) for 7 days to generate a cell population stably expressing gD. Once the non-transduced cells were killed by the puromycin, the medium was replaced with medium containing 1 μg/ml of puromycin and allowed to proliferate for 2 weeks. The pLenti-gD Puro transduced cells (TZM-bl-gD or 293-gD) were immunostained with anti-gD mouse monoclonal antibody (Santa Cruz, #sc21719**)** and a secondary rabbit anti-mouse antibody tagged with Alexa 594 (Abcam, #150128). A cell sorter was used to isolate gD expressing cells. The same cell line was immunostained with an irrelevant primary antibody and a secondary rabbit anti-mouse antibody tagged with Alexa 594 was used as a control. The pLenti CMV EGFP Puro (658-5) transduced cells were also selected with puromycin and sorted for EGFP expressing cells. This cell line (TZM-bl-EGFP and 293-EGFP) were used as our control for infectivity studies.

For infectivity assays, we used VSV-G protein pseudotyped NL4-3 virus. 293 cells plated in T-25 flasks were co-transfected with pNL4-3 and a plasmid expressing VSV-G using Thermofisher Turbofect reagent according to the manufacturer’s protocol. Virus was harvested at 48 h post-transfection and titrated for p24 levels. Equivalent levels of VSV-G pseudotyped NL4-3 (M.O.I. of 0.5) were used to inoculate 293-gD, 293-EGFP, TZM-bl-gD or TZM-bl-EGFP cells in 6-well culture plates. At 48 h, post-inoculation the culture medium was collected and the levels of p24 determined using p24 ELISA kits and infectious virus released determined using TZM-bl assays.

## Conclusions

The HSV-1 gD and gH/gL proteins restricted the release of infectious HIV-1 from cells. The mechanism by which HSV-1 gD restricted HIV-1 was not due to interference in the biosynthesis and transport of HIV-1 proteins but rather involved preventing the incorporation of the HIV-1 gp120/gp41 into virus particles. Cell lines expressing gD were also capable of restricting the release of infectious HIV-1. The gD protein may be useful in deciphering the mechanisms of gp120/gp41 incorporation into virus particles.

## Additional files

**Additional file 1: Figure S1.** The HSV-1 gD is expressed at similar levels in transfected and HSV-1 infected cells. 293 cells were transfected with the vector expressing HSV-1 gD or inoculated with HSV-1 (F strain) at an multiplicity of infection of 0.1. Cells were incubated for 30 h, starved in medium lacking methionine and cysteine and radiolabeled with ^35^S-methionine/cysteine for 16 h. The medium was removed, washed in PBS and cell lysed in RIPA buffer. The lysate was centrifuged to remove the nuclei followed by addition of a monoclonal antibody directed against gD (upper panel) or β-actin (lower panel). The immunoprecipitates were collected by incubation with protein A-Sepharose beads at 4C. The beads were washed three times with RIPA buffer and boiled in sample reducing buffer. The proteins separated by SDS-PAGE (10% gel), and proteins visualized using standard radiographic techniques. The immunoprecipitation results show that cells transfected with the vector expressing gD had lower levels of gD than cells infected with HSV-1.

**Additional file 2: Figure S2.** HSV-1 gB does not significantly affect the processing of HIV-1 Gag and gp160. 293 cells were co-transfected with empty pcDNA3.1(+) vector or one expressing gB and pNL4-3. At 24 h, cells were starved in medium lacking methionine/cysteine for 2 h followed by radiolabeling cultures with ^35^S-methionine/cysteine. The radiolabel was removed and washed three times in medium containing 100 × methionine/cysteine and chased in the same medium for 0, 1, 3, and 6 h. The culture medium was harvested, and cell lysates prepared as described in the Materials and Methods. HIV-1 Env and Gag proteins and HSV-1 gB were immunoprecipitated with appropriate antibodies. The immunoprecipitates were collected on protein-A-Sepharose, washed, and boiled in sample reducing buffer. The proteins were separated on 7.5% SDS gels and visualized using standard radiographic techniques. **a**, **b** HIV-1 proteins immunoprecipitated from the cell lysates (**a**) and culture medium (**b**) of cells co-transfected cells with a vector expressing gB and pNL4-3. Panels C and D HSV-1 gB protein immunoprecipitated from the cell lysates (**c**) and culture medium (**d**) of cells co-transfected with a vector expressing gB and pNL4-3.

**Additional file 3: Figure S3.** The HIV-1 gp41 is not observed in HIV-1 virus particles in the presence of HSV-1 gD. 293 cells were co-transfected with either empty pcDNA3.1(+) vector, pcDNA3.1(+) and pNL4-3Δ*env*, pcDNA3.1(+) and pNL4-3, or with a vector expressing gD and pNL4-3. At 30 h, the cells were starved for methionine/cysteine for 2 h and then radiolabeled for with ^35^S-methionine/cysteine for 16 h. At 48 h post-transfection, the cell culture medium was harvested and subjected to low speed centrifugation to remove cellular debris. The resulting supernatant was layered on a 20% sucrose cushion and subjected to ultracentrifugation to pellet viral particles as described in the experimental procedures. The pelleted virus was harvested, resuspended in RIPA buffer and used in immunoprecipitation analysis using appropriate antibodies to immunoprecipitate HIV-1 gp41 and gp160 (**a**, **b**) or HIV-1 proteins (Env, p55, and p24) (**c**, **d**). The immunoprecipitates were collected on protein-A-Sepharose, washed, and boiled in sample reducing buffer. The proteins were separated on SDS-PAGE and visualized using standard radiographic techniques. **a** HIV-1 gp41 immunoprecipitated from the culture medium prior to and after pelleting virus through a sucrose cushion by ultracentrifugation. **b** HIV-1 gp41 containing proteins (gp160 and gp41) immunoprecipitated from cell lysates. **c** HIV-1 proteins immunoprecipitated from the culture medium prior to and after pelleting virus through a sucrose cushion by ultracentrifugation. **d** HIV-1 proteins (Env, p55, and p24) immunoprecipitated from cell lysates.

**Additional file 4: Figure S4.** Sucrose density gradient centrifugation purification of virus reveals the gp120 is incorporated in viral particles in the presence of HSV-1 gB. 293 cells were co-transfected with either empty pcDNA3.1(+) vector and pNL4-3 or a vector expressing gB and pNL4-3. At 30 h, the cells were starved for methionine/cysteine, radiolabeled and the culture medium harvested at 48 h post-transfection. Following low speed centrifugation, the culture supernatants were layered onto a 20% sucrose cushion and virus pelleted by ultracentrifugation. The pelleted virus resuspended in DMEM without serum and layered on a discontinuous 20–60% sucrose gradient. The virus was subjected to ultracentrifugation for 20 h, 12 fractions were collected, and subjected to immunoprecipitation analysis using appropriate antibodies against HIV-1 p24 and gp120/gp41, or HSV-1 gB. The immunoprecipitates were collected on protein-A-Sepharose, washed, and boiled in sample reducing buffer. The proteins were separated on SDS-PAGE and visualized using standard radiographic techniques. **a** Immunoprecipitation of HIV-1 proteins from gradient fractions of cells co-transfected with a vector expressing gB and pNL4-3. **b** Immunoprecipitation of HSV-1 gB from gradient fractions of cells co-transfected with a vector expressing gB and pNL4-3.

## Abbreviations

HIV-1: human immunodeficiency virus type 1
HSV-1: herpes simplex virus type 1
