# Supplementary figures and images for: Analysis of herpes simplex type 1 gB, gD, and gH/gL on production of infectious HIV-1: HSV-1 gD restricts HIV-1 by exclusion of HIV-1 Env from maturing viral particles
Source: Retrovirology. 2019 Apr 2;16:9. doi: 10.1186/s12977-019-0470-5 (PMC6444546; doi:10.1186/s12977-019-0470-5)

## Slide 1
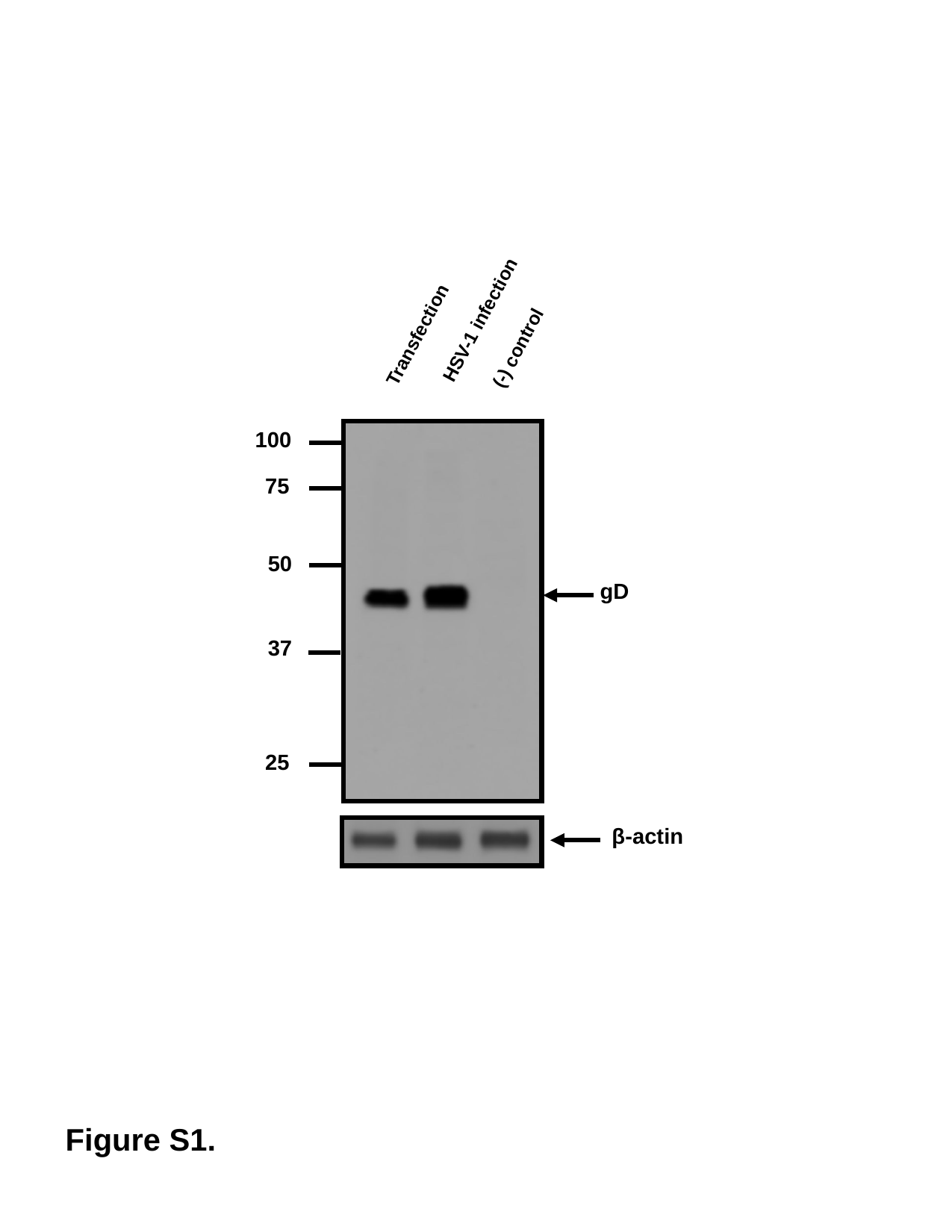

HSV-1 infection
Transfection
(-) control
100
75
50
gD
37
25
β-actin
Figure S1.

Supplement: Supplementary file 1 — Additional file 1: Figure S1. The HSV-1 gD is expressed at similar levels in transfected and HSV-1 infected cells. 293 cells were transfected with the vector expressing HSV-1 gD or inoculated with HSV-1 (F strain) at an multiplicity of infection of 0.1. Cells were incubated for 30 h, starved in medium lacking methionine and cysteine and radiolabeled with 35S-methionine/cysteine for 16 h. The medium was removed, washed in PBS and cell lysed in RIPA buffer. The lysate was centrifuged to remove the nuclei followed by addition of a monoclonal antibody directed against gD (upper panel) or β-actin (lower panel). The immunoprecipitates were collected by incubation with protein A-Sepharose beads at 4C. The beads were washed three times with RIPA buffer and boiled in sample reducing buffer. The proteins separated by SDS-PAGE (10% gel), and proteins visualized using standard radiographic techniques. The immunoprecipitation results show that cells transfected with the vector expressing gD had lower levels of gD than cells infected with HSV-1. [file 12977_2019_470_MOESM1_ESM.pptx]

## Slide 1
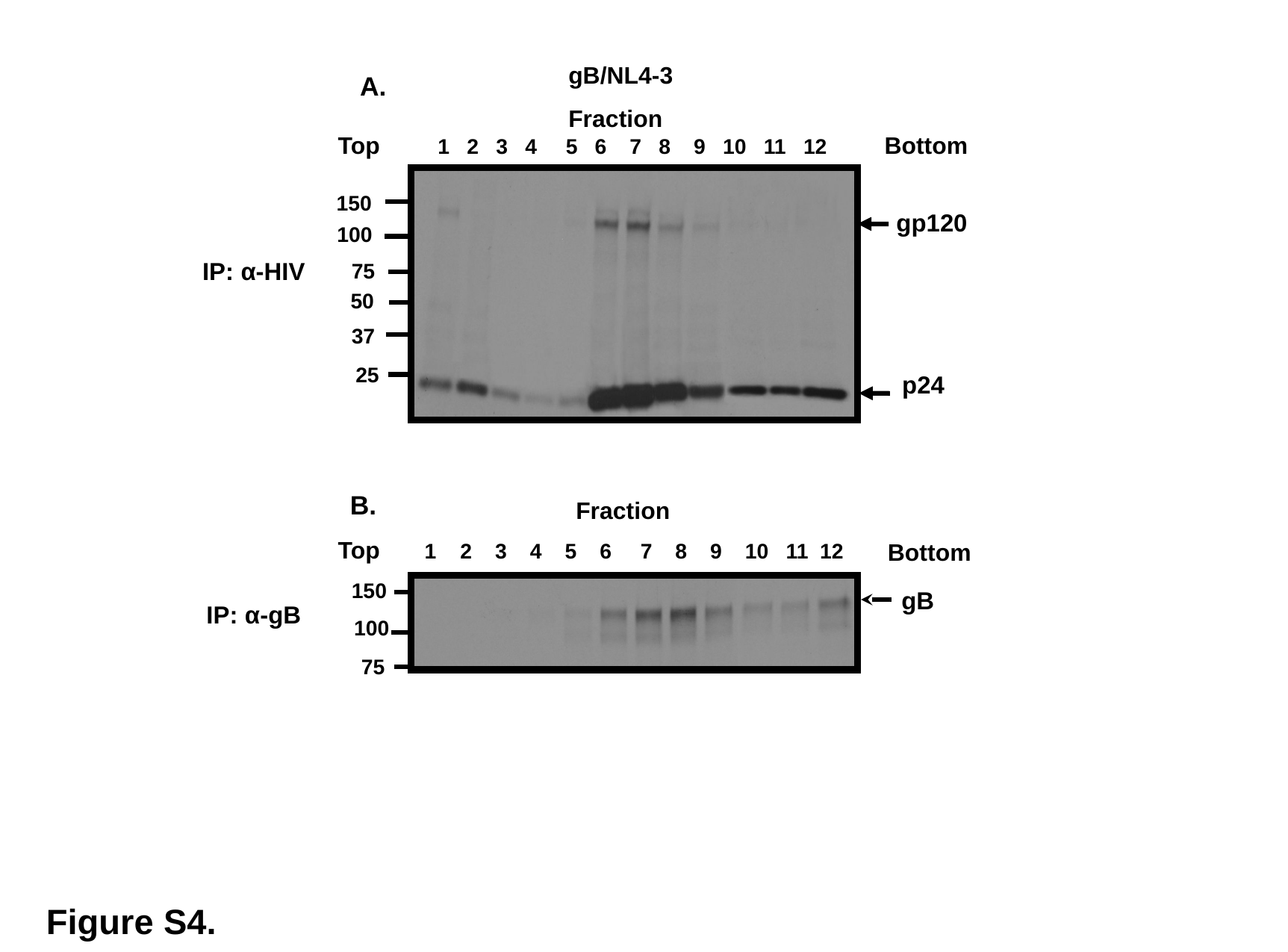

gB/NL4-3
A.
Fraction
Bottom
Top
1 2 3 4 5 6 7 8 9 10 11 12
150
gp120
100
IP: α-HIV
75
50
37
25
p24
B.
Fraction
Top
Bottom
2 3 4 5 6 7 8 9 10 11 12
150
gB
IP: α-gB
100
75
Figure S4.

Supplement: Supplementary file 4 — Additional file 4: Figure S4. Sucrose density gradient centrifugation purification of virus reveals the gp120 is incorporated in viral particles in the presence of HSV-1 gB. 293 cells were co-transfected with either empty pcDNA3.1(+) vector and pNL4-3 or a vector expressing gB and pNL4-3. At 30 h, the cells were starved for methionine/cysteine, radiolabeled and the culture medium harvested at 48 h post-transfection. Following low speed centrifugation, the culture supernatants were layered onto a 20% sucrose cushion and virus pelleted by ultracentrifugation. The pelleted virus resuspended in DMEM without serum and layered on a discontinuous 20–60% sucrose gradient. The virus was subjected to ultracentrifugation for 20 h, 12 fractions were collected, and subjected to immunoprecipitation analysis using appropriate antibodies against HIV-1 p24 and gp120/gp41, or HSV-1 gB. The immunoprecipitates were collected on protein-A-Sepharose, washed, and boiled in sample reducing buffer. The proteins were separated on SDS-PAGE and visualized using standard radiographic techniques. a Immunoprecipitation of HIV-1 proteins from gradient fractions of cells co-transfected with a vector expressing gB and pNL4-3. b Immunoprecipitation of HSV-1 gB from gradient fractions of cells co-transfected with a vector expressing gB and pNL4-3. [file 12977_2019_470_MOESM4_ESM.pptx]
