# Supplementary material for: Analysis of herpes simplex type 1 gB, gD, and gH/gL on production of infectious HIV-1: HSV-1 gD restricts HIV-1 by exclusion of HIV-1 Env from maturing viral particles
Source: Retrovirology. 2019 Apr 2;16:9. doi: 10.1186/s12977-019-0470-5 (PMC6444546; doi:10.1186/s12977-019-0470-5)
Supplement: Supplementary file 2 — Additional file 2: Figure S2. HSV-1 gB does not significantly affect the processing of HIV-1 Gag and gp160. 293 cells were co-transfected with empty pcDNA3.1(+) vector or one expressing gB and pNL4-3. At 24 h, cells were starved in medium lacking methionine/cysteine for 2 h followed by radiolabeling cultures with 35S-methionine/cysteine. The radiolabel was removed and washed three times in medium containing 100 × methionine/cysteine and chased in the same medium for 0, 1, 3, and 6 h. The culture medium was harvested, and cell lysates prepared as described in the Materials and Methods. HIV-1 Env and Gag proteins and HSV-1 gB were immunoprecipitated with appropriate antibodies. The immunoprecipitates were collected on protein-A-Sepharose, washed, and boiled in sample reducing buffer. The proteins were separated on 7.5% SDS gels and visualized using standard radiographic techniques. a, b HIV-1 proteins immunoprecipitated from the cell lysates (a) and culture medium (b) of cells co-transfected cells with a vector expressing gB and pNL4-3. Panels C and D HSV-1 gB protein immunoprecipitated from the cell lysates (c) and culture medium (d) of cells co-transfected with a vector expressing gB and pNL4-3. [file 12977_2019_470_MOESM2_ESM.pptx]

## Slide 1
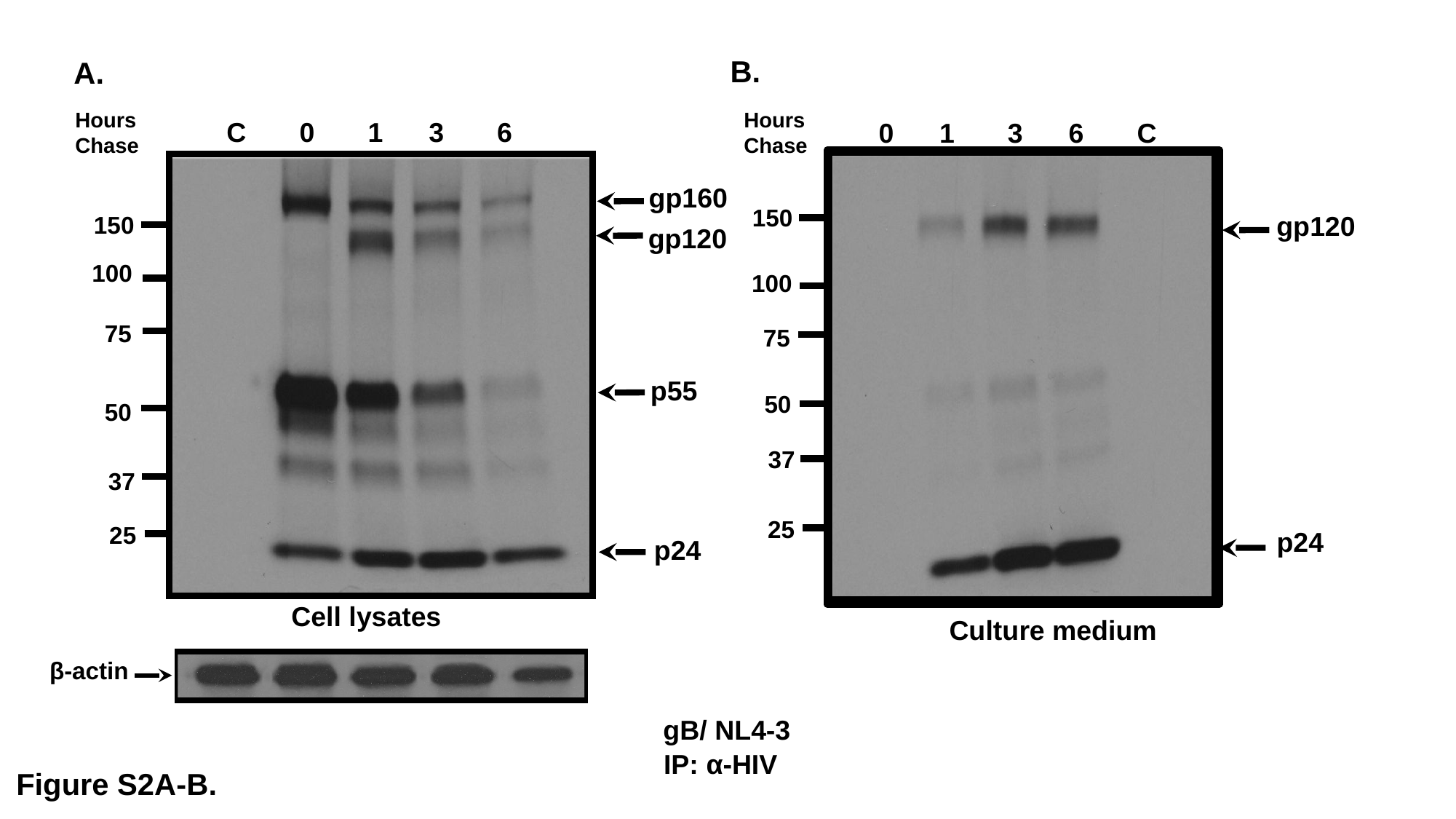

B.
A.
Hours
Chase
 C 0 1 3 6
gp160
150
gp120
100
75
p55
50
37
25
p24
Hours
Chase
 0 1 3 6 C
150
gp120
100
75
50
37
25
p24
Cell lysates
Culture medium
β-actin
gB/ NL4-3
IP: α-HIV
Figure S2A-B.

## Slide 2
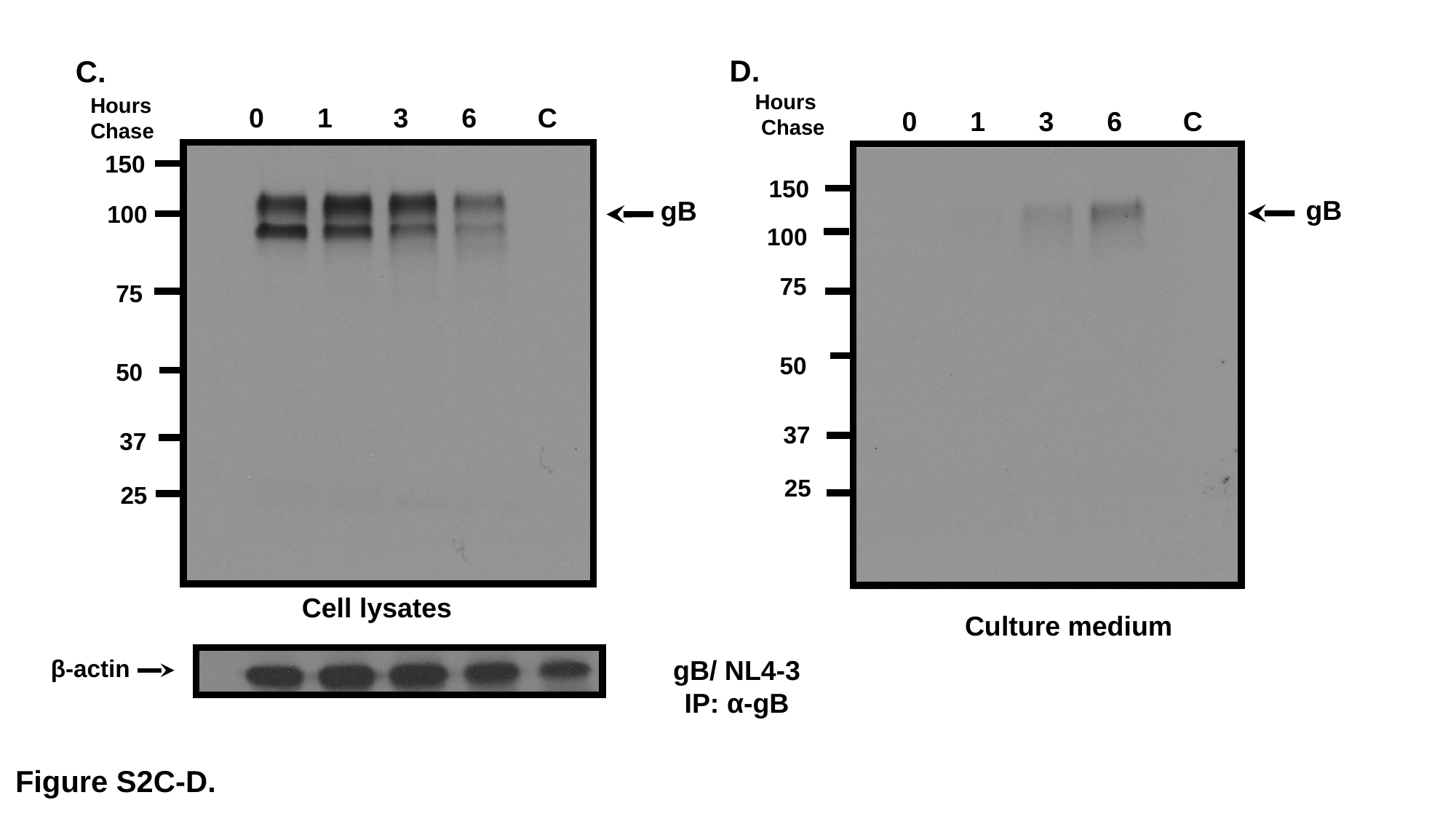

D.
C.
Hours
 Chase
 0 1 3 6 C
150
gB
100
75
50
37
25
Hours
Chase
 0 1 3 6 C
150
gB
100
75
50
37
25
Cell lysates
Culture medium
β-actin
gB/ NL4-3
IP: α-gB
Figure S2C-D.
