# Supplementary material for: Analysis of herpes simplex type 1 gB, gD, and gH/gL on production of infectious HIV-1: HSV-1 gD restricts HIV-1 by exclusion of HIV-1 Env from maturing viral particles
Source: Retrovirology. 2019 Apr 2;16:9. doi: 10.1186/s12977-019-0470-5 (PMC6444546; doi:10.1186/s12977-019-0470-5)
Supplement: Supplementary file 3 — Additional file 3: Figure S3. The HIV-1 gp41 is not observed in HIV-1 virus particles in the presence of HSV-1 gD. 293 cells were co-transfected with either empty pcDNA3.1(+) vector, pcDNA3.1(+) and pNL4-3Δenv, pcDNA3.1(+) and pNL4-3, or with a vector expressing gD and pNL4-3. At 30 h, the cells were starved for methionine/cysteine for 2 h and then radiolabeled for with 35S-methionine/cysteine for 16 h. At 48 h post-transfection, the cell culture medium was harvested and subjected to low speed centrifugation to remove cellular debris. The resulting supernatant was layered on a 20% sucrose cushion and subjected to ultracentrifugation to pellet viral particles as described in the experimental procedures. The pelleted virus was harvested, resuspended in RIPA buffer and used in immunoprecipitation analysis using appropriate antibodies to immunoprecipitate HIV-1 gp41 and gp160 (a, b) or HIV-1 proteins (Env, p55, and p24) (c, d). The immunoprecipitates were collected on protein-A-Sepharose, washed, and boiled in sample reducing buffer. The proteins were separated on SDS-PAGE and visualized using standard radiographic techniques. a HIV-1 gp41 immunoprecipitated from the culture medium prior to and after pelleting virus through a sucrose cushion by ultracentrifugation. b HIV-1 gp41 containing proteins (gp160 and gp41) immunoprecipitated from cell lysates. c HIV-1 proteins immunoprecipitated from the culture medium prior to and after pelleting virus through a sucrose cushion by ultracentrifugation. d HIV-1 proteins (Env, p55, and p24) immunoprecipitated from cell lysates. [file 12977_2019_470_MOESM3_ESM.pptx]

## Slide 1
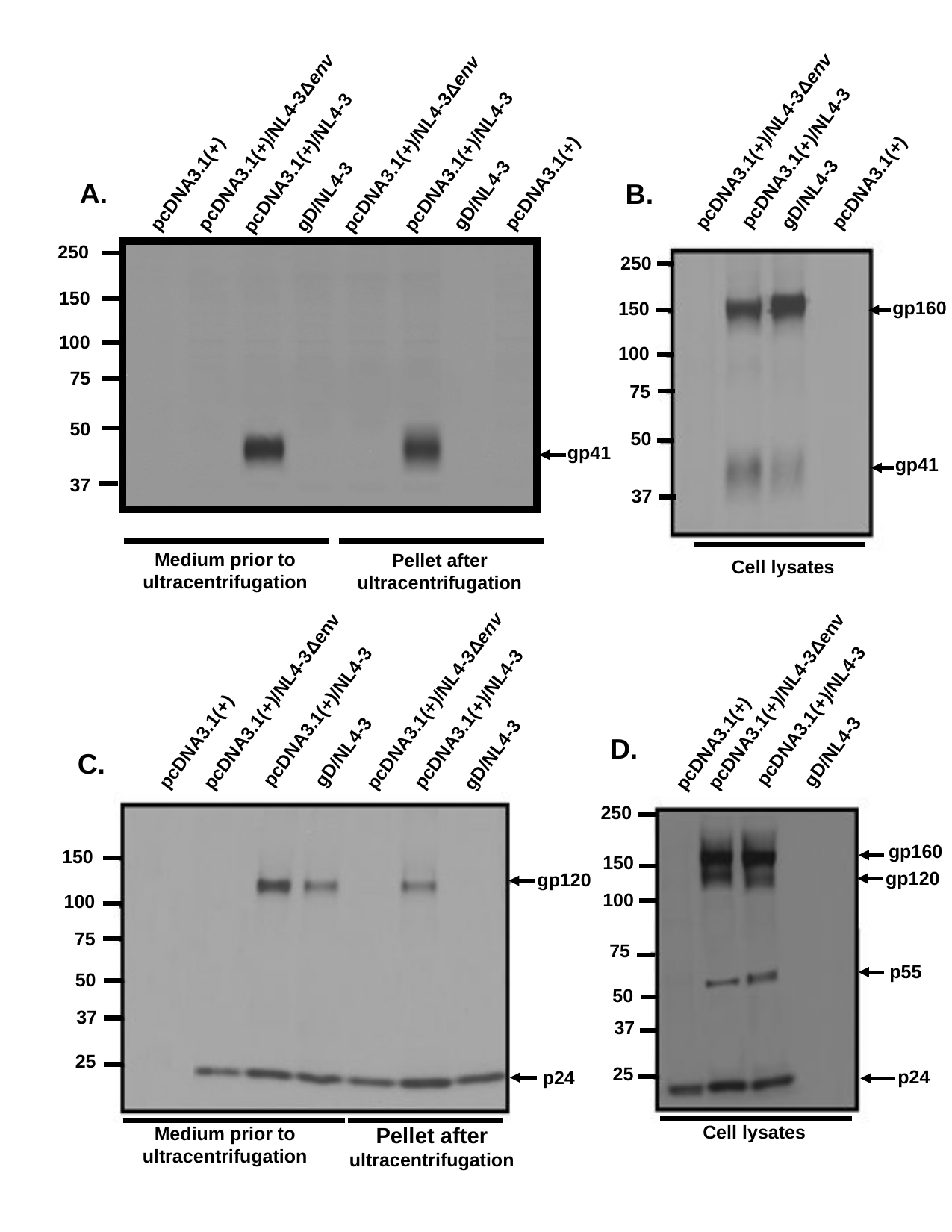

pcDNA3.1(+)/NL4-3Δenv
pcDNA3.1(+)/NL4-3Δenv
pcDNA3.1(+)/NL4-3Δenv
pcDNA3.1(+)/NL4-3
pcDNA3.1(+)/NL4-3
pcDNA3.1(+)/NL4-3
pcDNA3.1(+)
pcDNA3.1(+)
pcDNA3.1(+)
A.
B.
gD/NL4-3
gD/NL4-3
gD/NL4-3
250
250
150
gp160
150
100
100
75
75
50
50
gp41
gp41
37
37
Medium prior to
ultracentrifugation
Pellet after
ultracentrifugation
Cell lysates
pcDNA3.1(+)/NL4-3Δenv
pcDNA3.1(+)/NL4-3Δenv
pcDNA3.1(+)/NL4-3Δenv
pcDNA3.1(+)/NL4-3
pcDNA3.1(+)/NL4-3
pcDNA3.1(+)/NL4-3
pcDNA3.1(+)
pcDNA3.1(+)
D.
gD/NL4-3
gD/NL4-3
gD/NL4-3
C.
250
gp160
150
150
gp120
gp120
100
100
75
75
p55
50
50
37
37
25
25
p24
p24
Cell lysates
Pellet after
ultracentrifugation
Medium prior to
ultracentrifugation
